# Histological analysis of incremental markings and crown growth characteristics in mandibular first molars of the red fox, *Vulpes vulpes* (Canidae, Mammalia)

**DOI:** 10.1111/joa.70178

**Published:** 2026-05-18

**Authors:** Horst Kierdorf, Uwe Kierdorf

**Affiliations:** ^1^ Department of Biology University of Hildesheim Hildesheim Germany

**Keywords:** crown formation time, enamel, growth marks, laminations, periodicity

## Abstract

The present study analyzed incremental markings in the enamel of eight mandibular first molars (M_1_) of red foxes (*Vulpes vulpes*) and a single M_1_ of a grey wolf (*Canis lupus*). The most prominent enamel incremental markings in both species were laminations. Based on lamination counts, a mean crown formation time of 102 days (range 99–107) was reconstructed for the red fox M_1_. This finding fits well with the reported M_1_ emergence through the gum in this species at between days 105 and 119 after birth, indicating a daily periodicity of the laminations. Based on the nature of laminations as daily growth marks, we reconstructed different crown growth parameters in the red fox molars. Overall, the enamel daily secretion rate (DSR) increased from lowest values near the enamel–dentine junction (EDJ) to highest values near the outer enamel surface. The lowest mean value (6.8 μm/day) was recorded in the inner third of the lingual enamel from the cervical crown region, the highest mean (17.4 μm/day) in the outer third of the buccal enamel from the mid‐lateral crown region. While in the inner enamel third, DSR values were similar in buccal and lingual enamel, in the central and outer thirds, mean and median DSR values for buccal always exceeded those for lingual enamel. On the buccal crown side, linear enamel thickness and reconstructed ameloblast secretory lifespan were similar in the three lateral crown regions distinguished (upper, mid, lower) and only dropped in the cervical crown region. By contrast, lingually only the upper lateral crown region showed values similar to those on the buccal side, while the values in more cervical crown regions were always markedly lower than in the corresponding buccal crown regions. Enamel extension rate (EER) was highest in the upper third of the EDJ length, with mean values of 145 μm/day in buccal and 170 μm/day in lingual enamel. EER dropped to lowest values in the cervical third of the EDJ length, averaging 85 μm/day buccally and 97 μm/day lingually. The enamel formation front angle (EFFa) varied between 3.9° and 5.2°, with only slightly higher values in the lower compared with the upper crown portion. EER and the EFFa data indicate a very rapid crown elongation, which is reflected by the very steep inclination of the laminations throughout the enamel. Four to six subdaily growth increments were present between consecutive laminations, whereas long‐period enamel incremental markings were not discernible. Our findings indicate that previous studies on canid enamel misidentified the incremental markings by erroneously diagnosing daily laminations as long‐period markings (striae of Retzius) and subdaily prism cross‐striations as daily markings. In consequence, these studies reported too low DSRs for canid enamel and too long crown formation times for the analyzed teeth.

## Graphical abstract

This paper histologically analyzed incremental markings in dental enamel of red fox and grey wolf. Our results demonstrate that previous studies have misidentified these markings, which led to miscalculation of dental growth parameters.

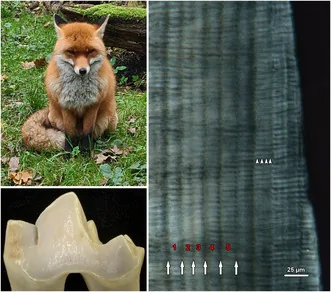

## 1 INTRODUCTION

The hard tissues (enamel, dentine, and cementum) of mammalian teeth are formed in an incremental way (Hillson, 2005, 2014; Hogg, 2018; Klevezal, 1996). Periodic oscillations in the secretory activity of the forming cells (ameloblasts, odontoblasts, cementoblasts) cause the occurrence of regular growth marks in the dental hard tissues (Hillson, 2005, 2014; Hogg, 2018; Klevezal, 1996; Newham & Naji, 2022). While cementum annulations record a yearly growth periodicity (Klevezal, 1996; Lieberman, 1993; Newham & Naji, 2022), the growth marks in enamel and dentine reflect short‐period (subdaily and daily) and long‐period (supradaily) periodicities (Bromage, 1991; Dean, 2000; Emken et al., 2021; Hillson, 2005, 2014; Hogg, 2018; Hullot et al., 2026; Kierdorf et al., 2013, 2014, 2019; Nacarino‐Meneses et al., 2025; Papakyrikos et al., 2020; Smith, 2006; Tafforeau et al., 2007).

The microscopic analysis of dental and skeletal growth marks enables the reconstruction of important life history traits in both extant and extinct mammals (Funston et al., 2022). Cementum growth marks can be used to determine age at death or season of death of individuals (Azorit et al., 2004, 2022; Klevezal, 1996; Lieberman, 1993; Veitschegger et al., 2019). Analysis of daily and supradaily growth marks in enamel and dentine allows to calculate crown and root formation times for the studied teeth and by this to pinpoint the timing of important life history events, such as weaning or the attainment of skeletal maturity (Calderon et al., 2026; Cuccu et al., 2025; Dean, 1987, 2000, 2006, 2017; Dirks et al., 2012; Emken et al., 2023; FitzGerald & Rose, 2008; Jordana & Köhler, 2011; Kierdorf et al., 2013, 2014, 2019; Nacarino‐Meneses et al., 2017, 2025; Orlandi‐Oliveras et al., 2019; Tafforeau et al., 2007). In addition, it is possible to assess the timing and duration of stress episodes that negatively affected tooth formation (Dirks et al., 2002; Kierdorf et al., 2021; Lemmers et al., 2021).

Historically, the study of enamel growth marks has focused on the teeth of primates, and the terminology used for the different structural enamel traits has been elaborated for this taxon (Boyde, 1989; Dean, 2006, 2017; Gustafson & Gustafson, 1967; Smith, 2008). In ground sections of primate enamel viewed in transmitted light, daily growth marks appear as alternating bright and dark bands (prism cross‐striations) oriented perpendicular to the long axis of the enamel prisms. A bright and a dark band represent a daily enamel growth increment (Boyde, 1989; Gustafson & Gustafson, 1967; Nanci, 2025). Supradaily (long‐period) enamel growth marks, indicating the consecutive position of the enamel forming front, are known as striae of Retzius or Retzius lines. Typically, these striae run at a certain angle to the course of the enamel prisms and in lateral enamel crop out in shallow furrows (perikyma grooves) at the outer enamel surface (OES) (Boyde, 1989; Hogg, 2018; Risnes, 1984). In cuspal enamel, the striae of Retzius are dome‐shaped over the dentine horn(s) and do not reach the OES (Boyde, 1989; Nanci, 2025; Risnes, 1998; Shellis, 1998). The time interval in days between consecutive striae of Retzius is called repeat interval and can be determined by counting the number of prism cross‐striations between them. In enamel of nonhuman primates, the repeat interval ranges between 1 and 11 days (Smith, 2008), while in human enamel between 6 and 12 days have been reported (Reid & Dean, 2006; Reid & Ferrell, 2006).

The enamel growth marks present in other mammalian taxa differ in some aspects from those in primate enamel. This is best documented for various extant and extinct ungulate taxa, including bovids (Cuccu et al., 2026; Jordana et al., 2014; Jordana & Köhler, 2011; Kierdorf et al., 2013), cervids (Cuccu et al., 2025, 2026; Iinuma et al., 2004; Jordana et al., 2014), giraffids (Nacarino‐Meneses et al., 2025), suids (Emken et al., 2021, 2023; Kierdorf et al., 2014, 2019; Okada & Mimura, 1940), equids (Calderon et al., 2026; Nacarino‐Meneses et al., 2017; Nacarino‐Meneses & Chinsamy, 2022; Orlandi‐Oliveras et al., 2019), and rhinocerotids (Tafforeau et al., 2007). For these taxa, it has been demonstrated that so‐called laminations represent the most prominent daily enamel growth marks. Laminations mark successive daily positions of the enamel forming front and therefore have the same orientation as the striae of Retzius. In contrast to primate enamel, where laminations are typically confined to prismless surface enamel (Kodaka et al., 1989, 1991; Ripa et al., 1966; Smith, 2006), in ungulate teeth they extend through the entire enamel layer. As has been demonstrated for equids (Calderon et al., 2026; Nacarino‐Meneses et al., 2017; Orlandi‐Oliveras et al., 2019), giraffids (Nacarino‐Meneses et al., 2025), and pigs (Emken et al., 2021; Kierdorf et al., 2019), a distinction between daily laminations and supradaily striae of Retzius is sometimes possible in ground sections, with the striae of Retzius being best visible in outer enamel. In cervical enamel of porcine molars, it was shown that, like in primate enamel, the striae of Retzius terminate in perikyma grooves at the OES (Kierdorf et al., 2019). By contrast, striae of Retzius are mostly not distinguishable from laminations in deeper enamel portions and in more cuspal crown regions where the inclination of the secretory front is steeper and the incremental markings reach the enamel surface at a very shallow angle.

In addition to daily laminations, also subdaily growth marks in the form of subdaily prism cross‐striations and/or subdaily laminations have been described in the enamel of sheep (Kierdorf et al., 2013), pigs (Emken et al., 2021; Kierdorf et al., 2019), giraffes (Nacarino‐Meneses et al., 2025), and equids (Calderon et al., 2026). In these studies, the number of subdaily increments between consecutive daily laminations typically ranged between two and five, thereby indicating variation in subdaily growth periodicities. In combination with the much higher daily secretion rate (DSR) of ungulate compared with primate enamel, this can cause a misidentification of enamel growth marks in ungulate teeth. Thus subdaily cross‐striations in ungulate enamel may be mistaken for daily cross‐striations and daily laminations for supradaily striae of Retzius. Such a misinterpretation of enamel incremental markings will lead to a miscalculation of dental growth parameters of ungulates. Several examples of such misidentification of enamel incremental markings in extant and extinct ungulate taxa have been identified and discussed by Kierdorf et al. (2013, 2014, 2019) and Nacarino‐Meneses et al. (2017). While in extant species such errors can be resolved by comparing histologically determined crown formation times (CFTs) with known eruption times of the respective tooth or radiographic information on its development, the situation is more difficult in extinct species for which such information is not available. However, as has recently been demonstrated by Hullot et al. (2026), the well‐established periodicity of enamel incremental markings of ungulate teeth may enable a proper characterization of crown growth parameters also in extinct taxa, even when they have no living relatives.

Compared to the situation in primates and ungulates, our current knowledge of the appearance and periodicity of enamel incremental markings in other mammalian taxa is scarce. As has been stressed by Hogg (2018), there is therefore an urgent need for more information on these dental traits in more mammalian taxa. This includes the order Carnivora, for which at present only very limited information on dental growth marks is available.

In an overview paper, Fukuhara (1959) published data for single teeth from various families of Carnivora (Felidae, Viveridae, Canidae, Ursidae, Otariidae, Phocidae). The reported DSRs ranged between 1.5 and 2.5 μm/day, while the reported width of single long‐period (Retzius) increments varied between 6 and 12 μm. For the enamel of domestic dogs (*Canis familiaris*), Fukuhara (1959) reported a DSR of 2.4 μm/day and a long‐period repeat interval of four. For the enamel of a maxillary fourth premolar (P^4^) of a wolf (*Canis lupus*) recovered from an archaeological site in the Czech Republic, Sazelova et al. (2020) present a DSR of 4.1 μm/day and a long‐period repeat interval of four. In their study on enamel histology of permanent canines from different domestic dog breeds, Hogg et al. (2018) report a long‐period repeat interval between four and six, but do not provide data on other dental growth parameters. The only value given by these authors is the total field width of the images that they used to illustrate incremental markings in enamel and dentine of the dog teeth. From this, it is possible to calculate a DSR of approximately 5 μm/day on the basis of the daily growth increments allegedly identified by these authors. By contrast, a much higher DSR of 17–19 μm/day was recently determined by Kierdorf et al. (2024) in outer lateral enamel of a cave bear (*Ursus spelaeus*) maxillary second molar (M^2^). The latter study further reported the presence of five subdaily growth increments between consecutive daily laminations in the cave bear enamel.

The conflicting statements regarding the nature of incremental markings in carnivoran enamel and the highly varying DSRs reported indicate the need for a clarification of the periodicity of enamel growth marks in this taxon. The aim of the present paper was to provide such a clarification, focusing on the teeth of the red fox (*Vulpes vulpes*), the most widespread of all wild canid species (Sillero‐Zubiri, 2009).

## 2 MATERIALS AND METHODS

### 2.1 Study sample

The study was performed on eight juvenile or young adult red foxes (five males, three females) and, in addition, one juvenile male grey wolf (*C. lupus*) (Table 1). The red foxes originated from Western Germany (federal state of North Rhine‐Westphalia) and had been collected during regular hunting operations. The grey wolf originated from Eastern Germany (federal state of Brandenburg) and had been killed in a car accident. For the study, we purposely selected red foxes with a completely formed permanent dentition that showed either no or only minor (individual #6) dental wear.

**TABLE 1:** Overview of the individuals from which first mandibular molars were analyzed.

Individual/species	Sex	Approximate age at death (months)
#2/*Vulpes vulpes*	Female	4–5
#3/*Vulpes vulpes*	Male	4–5
#4/*Vulpes vulpes*	Male	5–6
#6/*Vulpes vulpes*	Male	16–17
#7/*Vulpes vulpes*	Male	6–7
#8/*Vulpes vulpes*	Male	6–7
#9/*Vulpes vulpes*	Female	6–7
#10/*Vulpes vulpes*	Female	6–7
#1/*Canis lupus*	Male	11–12

*Note*: Age of the individuals was assessed based on their known date of death and an assumed birth date. The birth season of the red fox in Central Europe spans the period from mid‐March to mid‐April (Wandeler & Lüps, 1993), while births in grey wolves from Central Europe occur mainly in April and May (Peters, 1993).

The dental formula of both the red fox and the grey wolf is 3‐1‐4‐2/3‐1‐4‐3, with their permanent dentitions comprising 42 teeth. The permanent dentition is completed (all teeth fully erupted) at about 5 months of age in the red fox and at about 5–5.5 months in the grey wolf (Geiger et al., 2016; Peters, 1993; Wandeler & Lüps, 1993).

### 2.2 Preparation of histological ground sections

Following maceration, cleaning and drying of the skulls, the mandibular left first molars (M_1_) were extracted and freed from adherent soft tissues. The M_1_ is the largest cheek tooth in the permanent dentition of the red fox and grey wolf and part of the carnassial complex (P^4^/M_1_) that is typical for most Canidae and other carnivorans (Ungar, 2010). The extracted teeth were photographed with a digital microscope (VHX 7000, Keyence, Osaka, Japan) (Figure 1). They were then embedded in epoxy resin (Biodur® E12, Biodur Products, Heidelberg) and sectioned axio‐buccolingually close to the tip of the protoconid with a water‐cooled diamond disc. The section surfaces were manually smoothed and polished using a graded series of silicon carbide sandpapers (grits 600–3000), followed by polishing on a motorized rotary polisher (Labopol 5, Struers, Ballerup, Denmark) using a 3 μm diamond suspension and finally a 0.3 μm alumina slurry.

**FIGURE 1:**
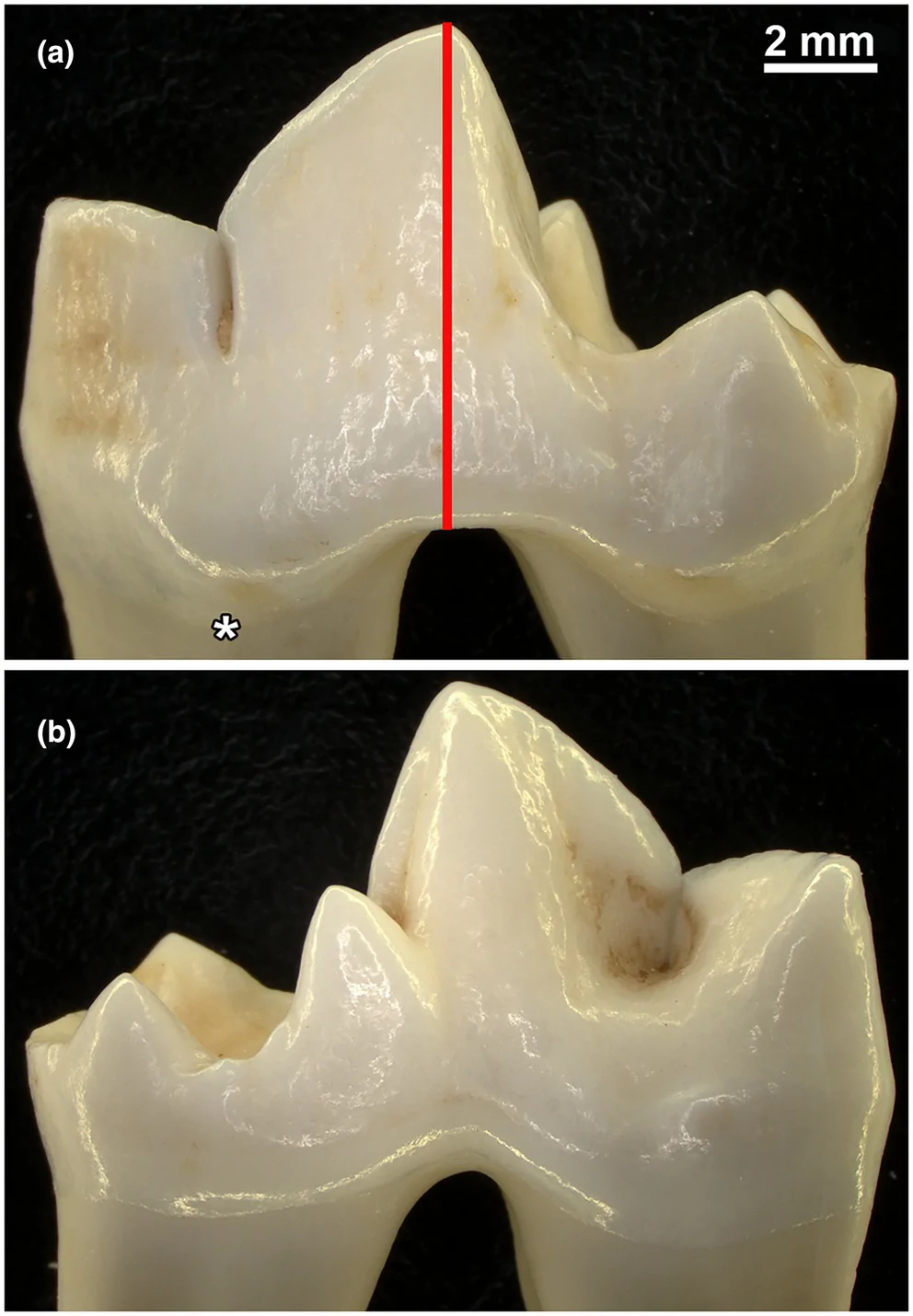
Unworn left M_1_ of a red fox (individual #2). (a) Buccal view, mesial to the left. Red line indicates the section plane running through the tip of the protoconid. Asterisk: Deepest (apical‐most) position of the crown‐root border in the tooth. (b) Lingual view, mesial to the right.

The polished sectional block surfaces of selected teeth were etched for 5 s with 5% (v/v) phosphoric acid, thoroughly rinsed with tap and distilled water, and air‐dried. The block surfaces were then examined in a scanning electron microscope (SEM Evo Ma 15, Carl Zeiss, Oberkochen, Germany) operated in a low vacuum mode at 20 kV using a backscattered electron (BSE) detector. Following acquisition of the BSE images, all mesial tooth halves were mounted on glass slides with the polished side facing the slide, using epoxy resin as glue. The mounted blocks were sectioned with the diamond disc to a thickness of approximately 200 μm. Thin ground sections of about 40–50 μm thickness were then produced by grinding with silicon carbide sandpapers and a final polishing step with a leather cloth and a dry polishing compound. The cover‐slipped ground sections were viewed and photographed with a Keyence VHX 7000 digital microscope equipped with a high‐performance 100×−1000× zoom lens and a Zeiss Axio Imager 2 microscope equipped with an Axiocam 503 color digital camera (Carl Zeiss, Oberkochen, Germany), using either plain transmitted light, transmitted light with phase‐contrast enhancement, or linearly polarized transmitted light with crossed polars.

Visibility of the different enamel incremental markings varied according to the imaging modalities and magnifications used in the microscopic analysis of the ground sections. As a consequence, it was difficult to demonstrate daily and subdaily markings with similar distinctness in the same image.

### 2.3 Analysis of dental growth parameters

All measurements were performed with the tools of the Fiji freeware software package of ImageJ (NIH, Bethesda, USA) on stitched images of the buccal and lingual enamel obtained in transmitted light with phase‐contrast enhancement, using a 10× magnification objective of the Axio Imager 2 microscope.

In the red fox M_1_s, the following dental growth parameters were determined based on the assumption that the regular enamel incremental markings (laminations) represent daily growth marks.

- *Crown formation time (CFT*, days) was established by first counting the total number of laminations present in buccal enamel. However, the vertical section plane running through the tip of the protoconid did not include the most cervically located enamel portion, that is, the plane did not run through the deepest (most apical) point of the crown‐root border (CRB) (Figure 1). Therefore, the distance between the location of the CRB in the section plane and the most apical position of the CRB, located on the buccal side of the tooth, was determined and divided by the enamel extension rate (EER) established for the 10th decile of the enamel–dentine junction (EDJ) length in the section plane. By this, we obtained a value for the number of days that had to be added to the number of laminations recorded in the ground sections in order to determine the CFT.
- *Daily secretion rate (DSR*, μm/day) was recorded at four defined positions along the vertical crown axis (upper lateral, mid‐lateral, lower lateral, and cervical crown regions) on the buccal and lingual crown sides (Figure 2). The upper lateral position was located at 15% of the total EDJ length below the cusp tip, and the cervical position at about 15% of the respective EDJ length above the CRB. The mid‐lateral and lower lateral positions were located, respectively, at 1/3 and 2/3 of the distance along the EDJ between the upper lateral and cervical positions. DSR was established at each of the four positions for the inner, central, and outer third of the enamel layer by measuring the distance between two laminations along a prism course (Figure 3a) and dividing this value by the number of daily increments (lamination intervals) between them (Figure 3b).
- *Linear enamel thickness* (LET, μm) was measured as the distance between EDJ and the OES perpendicular to the EDJ at the four positions (upper lateral, mid‐lateral, lower lateral, cervical crown regions) for the buccal and lingual crown sides.
- *Ameloblast secretory lifespan* (ASL, days), that is, the duration of appositional enamel matrix deposition by the secretory ameloblasts, was determined by counting the number of laminations intersecting the reconstructed path of an individual enamel prism from the EDJ to the OES in the four crown regions (Figure 3a).
- *Enamel extension rate (EER*, μm/day) was calculated for thirds of the complete EDJ length (first third = cuspal third) on the buccal and lingual crown sides. For this, the length of the respective EDJ stretch was divided by the number of laminations meeting the EDJ in the respective third. The EER reflects the rate at which ameloblasts enter the secretory stage of amelogenesis (Shellis, 1984, 1998).
- *Enamel formation front angle* (EEFa, degrees), that is, the angle between the EDJ and the laminations (marking the enamel secretory front) was determined at two positions of the buccal and lingual crown side. The upper position was located at 30% of the EDJ length below the tip of the dentine horn. The lower position was located at 30% of the EDJ length above the CRB. The EFFa is related to the number of secretory ameloblasts simultaneously active along the enamel forming front and closely linked to the EER (the higher the EER the smaller the EFFa).

**FIGURE 2:**
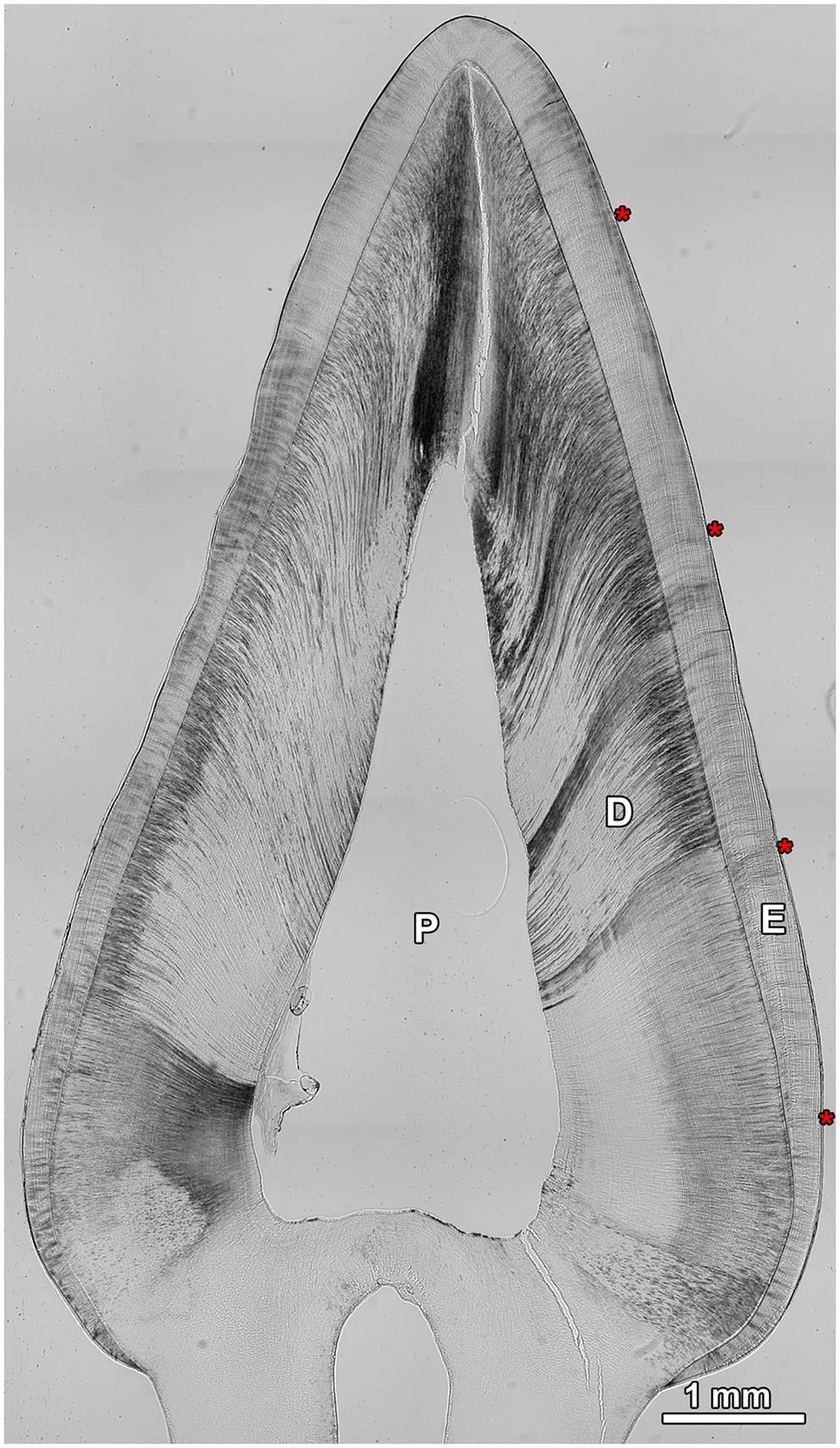
Micrograph of buccolingual ground section of the left M_1_ of a red fox (individual #6), buccal to the right. Section viewed in plain transmitted light. Red asterisks: Approximate location of measurement sites (from top to bottom: Upper lateral, mid‐lateral, lower lateral and cervical crown region). Note the difference in thickness between buccal and lingual enamel. D, dentine; E, enamel; P, pulp cavity.

**FIGURE 3:**
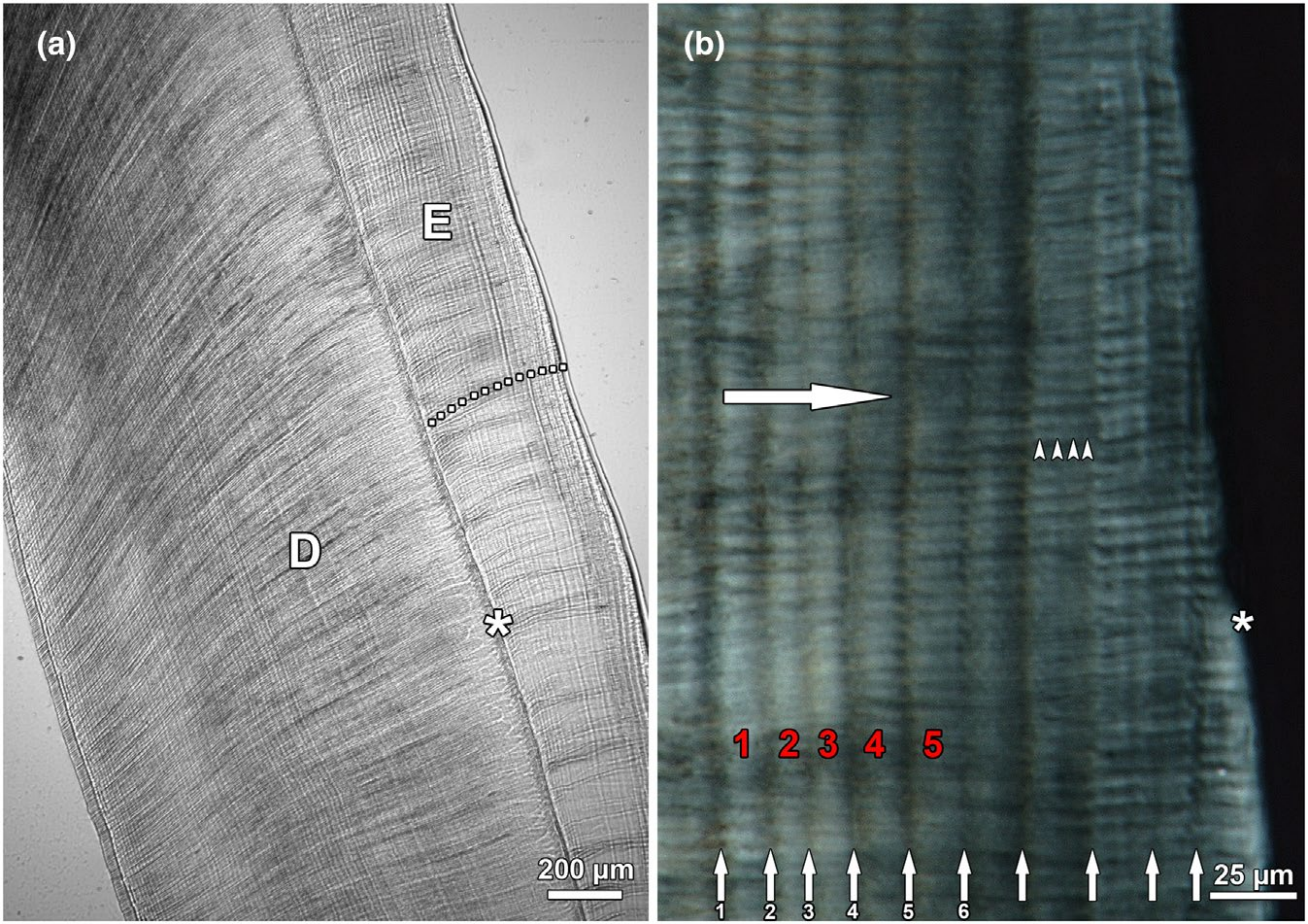
Micrographs of buccolingual ground sections of the left M_1_ of two red foxes, cuspal to the top. (a) Mid‐lateral buccal crown region of M_1_ of individual #3. Section viewed in plain transmitted light. Enamel (E) and dentine (D) exhibit prominent daily incremental markings (laminations in enamel and von Ebner lines in dentine). Asterisk: Enamel–dentine junction (EDJ). Dotted line: Approximate course of an enamel prism from the EDJ to the outer enamel surface (OES). (b) Outer buccal enamel of mid‐lateral crown region of M_1_ of individual #2. Section viewed in linearly polarized transmitted light with crossed polars. Small arrows mark 10 laminations, six of which are numbered. Red numbers indicate five daily growth increments between these six laminations. Mean width for the daily growth increments located between the innermost marked lamination (“1”) and the OES (asterisk) is about 17 μm. In places, four subdaily growth increments (arrowheads) are discernible between consecutive laminations. Large arrow: Prism direction. Note the very small angle between laminations and OES.

Due to partial damage of the ground section, for the wolf M_1_ only some data for DSR and LET could be obtained.

### 2.4 Statistics

All statistical tests were performed with the software package Statistica, version 13.3 (TIBCO, Palo Alto, USA), using non‐parametric tests for dependent samples. *p*‐values <0.05 were considered significant. The differences between two groups were assessed using the Wilcoxon matched pairs test. Differences among more than two groups were analyzed using Friedman ANOVA by ranks. If significance was indicated, this was followed by pairwise post‐hoc comparisons using Wilcoxon matched pairs tests. In order to control for type I error, we applied a Bonferroni correction of *p*‐values when performing multiple post‐hoc tests. In these cases, both nominal (unadjusted) and Bonferroni‐adjusted *p*‐values are reported.

## 3 RESULTS

### 3.1 Incremental markings, DSR and CFT

Microscopic inspection of the red fox M_1_ ground sections revealed the presence of regular incremental markings in both enamel (laminations) and dentine (von Ebner lines) (Figures 3, 4, 5). These incremental markings were regarded to reflect a daily growth rhythm, and all calculations of dental growth parameters were based on this assumption. Although some laminations appeared more prominent than others, a regular periodic pattern of morphologically more pronounced, long‐period incremental markings (striae of Retzius) was neither discernible in the ground sections (Figures 3, 4, 5) nor in the etched block surfaces viewed in the SEM (Figure 6). Also, no accentuated incremental marking that could qualify as a neonatal line (NNL) was recorded in the cuspal enamel of any of the analyzed red fox molars (Figure 5a).

A mean number of 88 laminations (range 85–93) were recorded in the enamel of the buccal crown side. To this number, we added 14 days to account for the cervical enamel portion not represented in the section (Figure 1). This resulted in a mean CFT of 102 days (range 99–107 days) for the red fox M_1_.

In buccal and lingual enamel, the spacing of consecutive laminations increased from the EDJ to the OES, indicating an increase in DSR from inner to outer enamel (Figure 4a). The lowest mean DSR value (6.8 μm/day) was present in lingual inner enamel of the cervical crown region, while the highest mean values were recorded for buccal outer enamel of the upper lateral (17.3 μm/day) and mid‐lateral (17.4 μm/day) crown regions (Figure 7; Table S1). Differences in DSR between the four crown regions were not significant in inner, central, and outer buccal enamel and in inner and outer lingual enamel, while a significant difference was found for the central third of the lingual enamel (Figure 7). Post‐hoc comparisons revealed significant differences between upper lateral and cervical (nominal *p*‐value = 0.028) and lower lateral and cervical enamel (nominal *p*‐value = 0.043) of the lingual central third, but significance was no longer given after performing Bonferroni correction (adjusted *p*‐values of 0.168 and 0.258, respectively).

**FIGURE 4:**
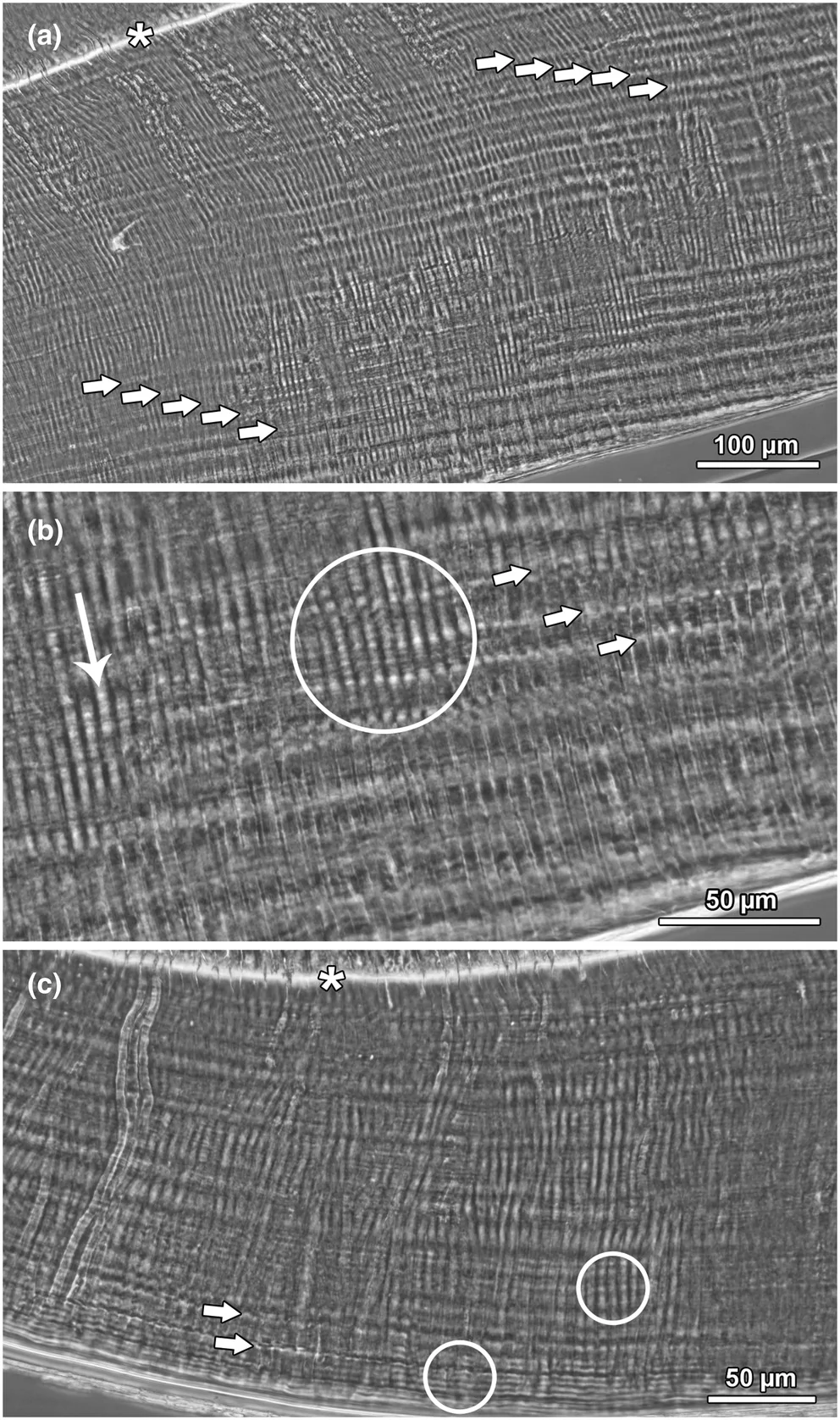
Micrographs of buccolingual ground sections of the left M_1_ of red foxes, sections viewed in transmitted light with phase‐contrast enhancement, cuspal to the right. (a) Buccal enamel (mid‐lateral crown region) of M_1_ of individual #2. Two groups of laminations are marked by arrows. Note the increase in spacing between laminations from inner to outer enamel. Asterisk: Enamel–dentine junction (EDJ). (b) Higher magnification of the outer zone of the enamel layer depicted in (a). White circle: Area where subdaily growth increments are discernible between consecutive laminations (short arrows). Subdaily increments appear as alternating bright and dark cross‐striations. Long arrow: Prism course oriented almost perpendicular to the laminations. (c) Cervical buccal enamel of M_1_ of individual #7. The inner white circle marks an enamel area where the subdaily growth increments appear broader (block‐like), the outer white circle marks a peripheral enamel area where the subdaily growth increments are markedly narrower, thereby indicating a lower daily secretion rate (DSR) in this enamel zone. Arrows: Two consecutive laminations in peripheral enamel. Asterisk: EDJ.

Both buccally and lingually, the differences in DSR among enamel areas (inner, central, outer third) were significant for all four crown regions (Table S2). While in pairwise comparisons of the thirds within a crown region, the nominal *p*‐values were all significant (<0.05), following Bonferroni correction significance was only maintained for the majority of comparisons for the buccal crown regions (Table S2). While DSRs for the inner third did not significantly differ between buccal and lingual enamel in all four crown regions (Wilcoxon matched pairs tests, all *p*‐values >0.2), in the central and outer thirds of the enamel layer, mean and median DSR values for buccal always exceeded those for lingual enamel (Table S1), the differences between the two crown sides being significant (Wilcoxon matched pairs tests, all *p*‐values <0.05).

The laminations followed a steeply inclined course from the EDJ to the OES, indicative of extended enamel secretory fronts (Figures 3a,b, 4a, and 6a). As a consequence of this steep inclination of the laminations, the zone of imbricational enamel reached nearly up to the crown tip, leaving only a very small area of cuspal enamel (Figure 5a). As a further consequence of their steep inclination, the laminations formed a very shallow angle with the OES (Figures 3a,b, 4a, and 6a). In lateral enamel, the inclination of the prisms was much lower than that of the laminations, and the prisms therefore crossed the laminations at a more or less right angle in central and outer enamel (Figures 3a,b, 4b, and 6). Between consecutive laminations, subdaily increments (*n* = 4–6) were discernible (Figures 3b, 4b,c, 5b, and 6b). In areas with high DSR values, the subdaily increments appeared broader (block‐like), while they were narrower (band‐like) in areas with lower DSR values (Figure 4b,c).

**FIGURE 5:**
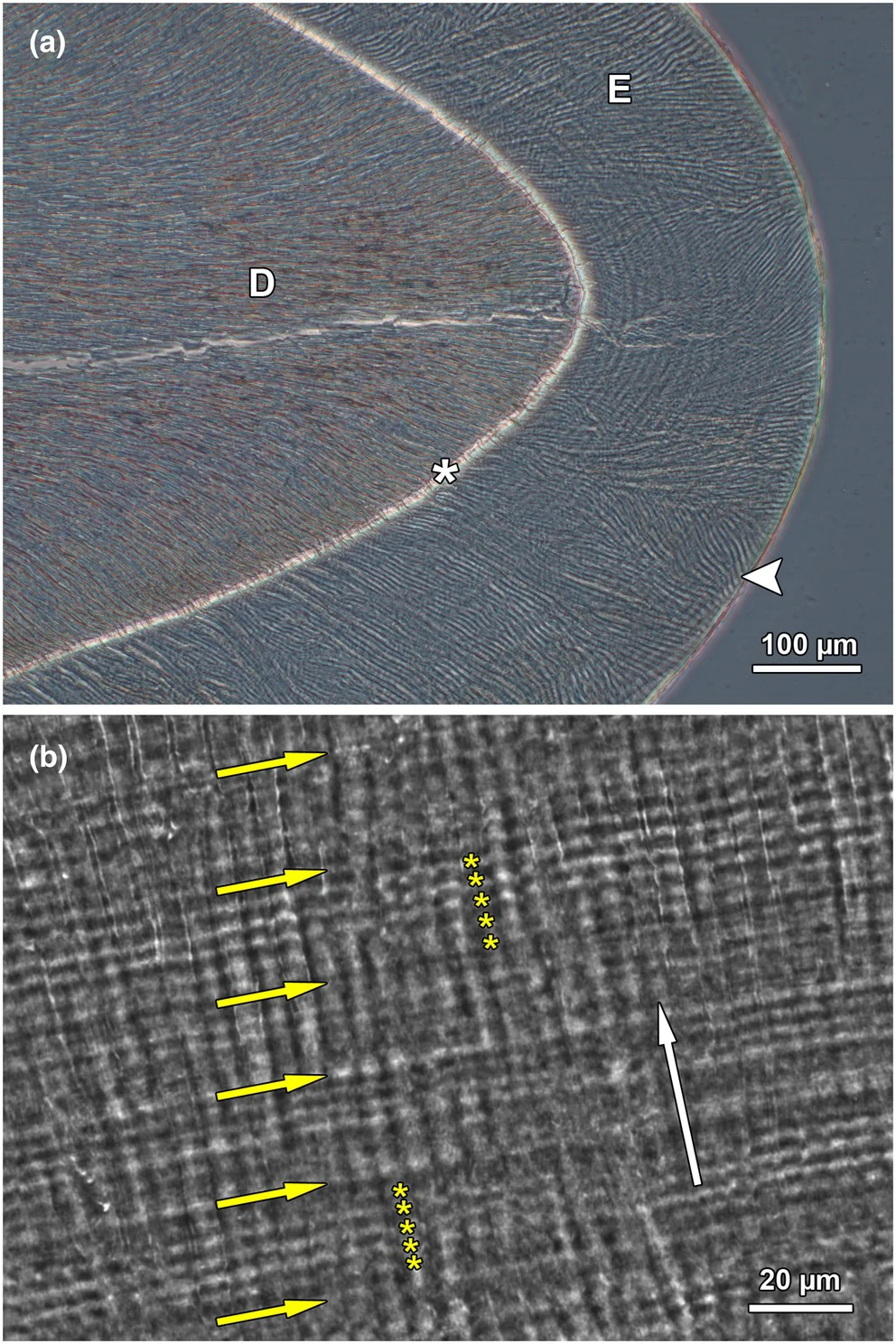
Micrographs of buccolingual ground sections of left M_1_ of red foxes. Sections viewed in transmitted light with phase‐contrast enhancement, cuspal to the right. (a) Cusp tip of M_1_ of individual #8, the cuspal‐most lamination of the imbricational enamel reaches the outer enamel surface (OES) close to the protoconid tip (arrowhead), leaving only a small area of cuspal enamel where laminations are dome‐shaped over the dentine horn. No accentuated incremental marking that could represent a neonatal line is discernible in enamel (E) or dentine (D). Asterisk: Enamel–dentine junction (EDJ). (b) Detail of outer buccal enamel in the mid‐lateral crown region of the M_1_ of individual #4. The yellow arrows mark six laminations, the yellow asterisks indicate five subdaily incremental markings between consecutive laminations, denoting presence of six subdaily growth increments. The white arrow indicates overall prism direction. Cuspal to the right, OES to the top of the image.

**FIGURE 6:**
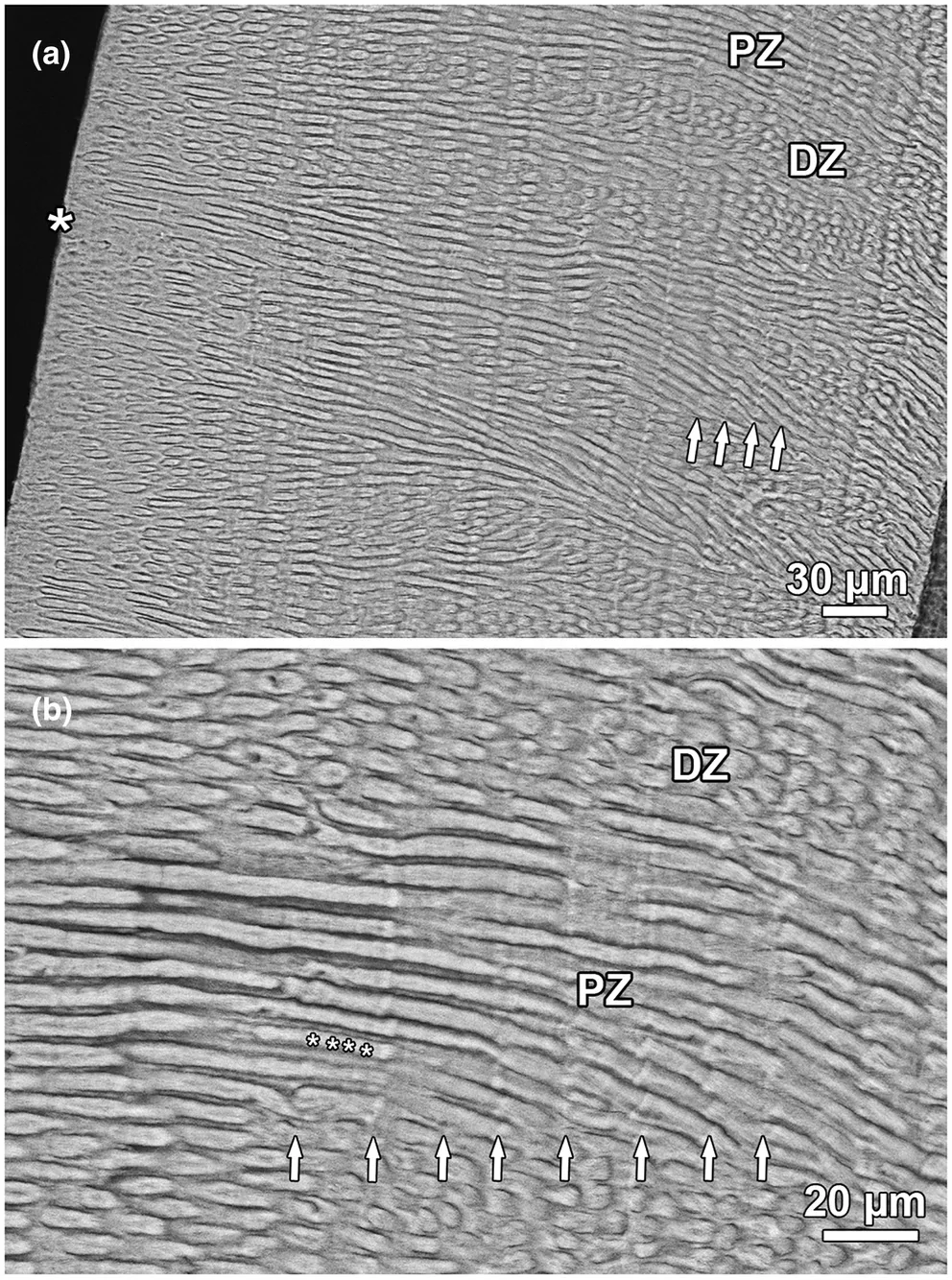
SEM‐backscattered electron (BSE) image of polished and etched sectional block surface showing buccal enamel in the cervical crown region of the left M_1_ of red fox individual #2, cuspal to the top, outer enamel surface (OES) to the left. (a) Prominent Hunter–Schreger bands indicating marked prism decussation in most of the enamel layer. DZ, diazone with more or less cross‐sectioned prisms; PZ, parazone with tangentially sectioned prisms. Only a small outermost enamel zone shows a radial prism orientation. The laminations (arrows) run at a steep course throughout the enamel. Asterisk: OES. (b) Higher magnification showing central to outer enamel. Laminations (arrows) are visible in both diazones (DZ) and parazones (PZ), whereas subdaily incremental markings (asterisks) are only discernible in the tangentially sectioned prims of the parazones.

**FIGURE 7:**
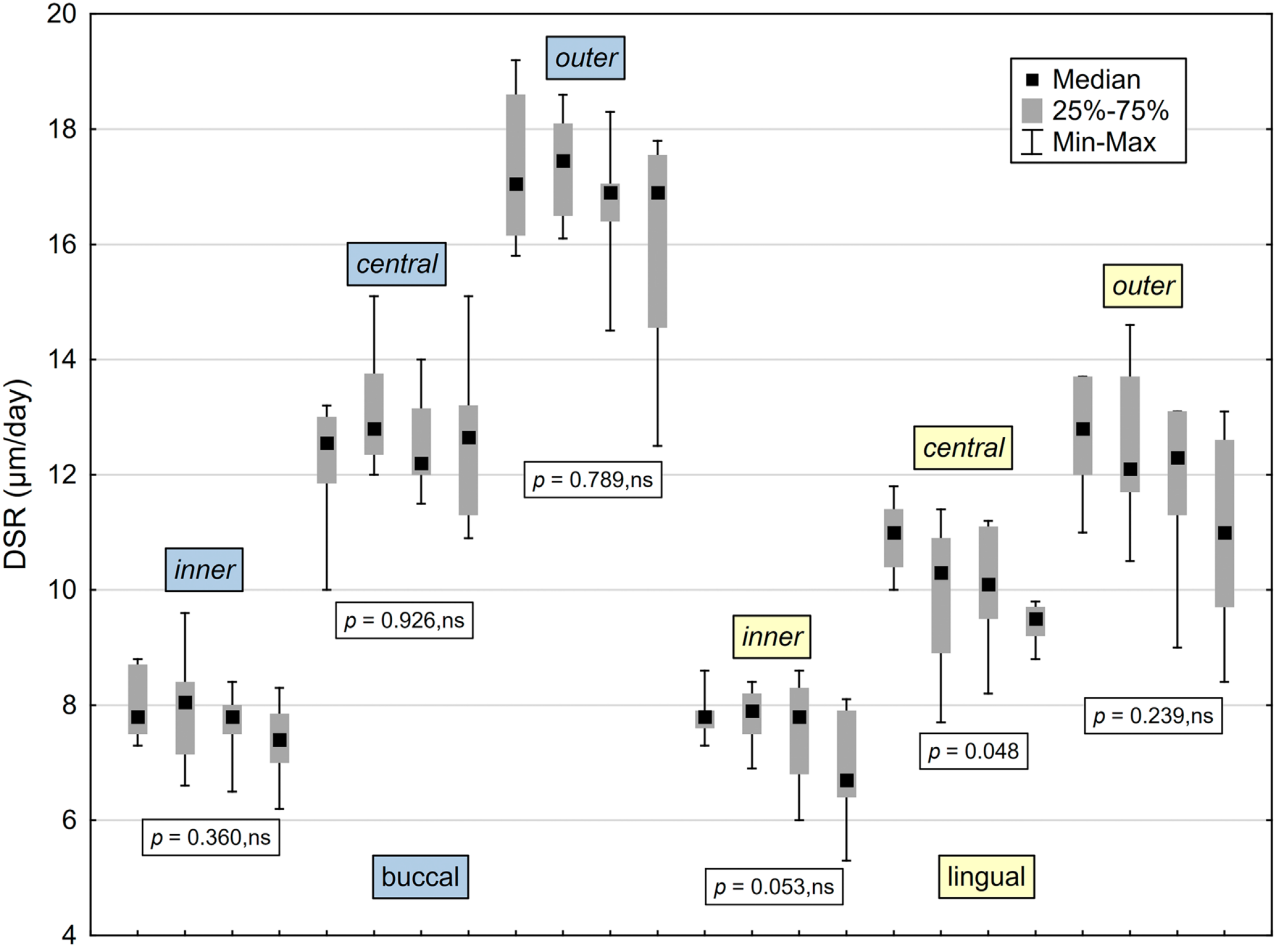
Daily enamel secretion rate (DSR) recorded in the three enamel zones (inner, central, outer) of the four analyzed crown regions of buccal and lingual enamel. Within each row, the sequence from left to right is upper lateral, mid‐lateral, lower lateral, and cervical crown region. The significance of the differences among these four regions in the three enamel zones is given (Friedman ANOVA by ranks). Except for the central enamel zone of the lingual side, the differences are not significant (ns).

**FIGURE 8:**
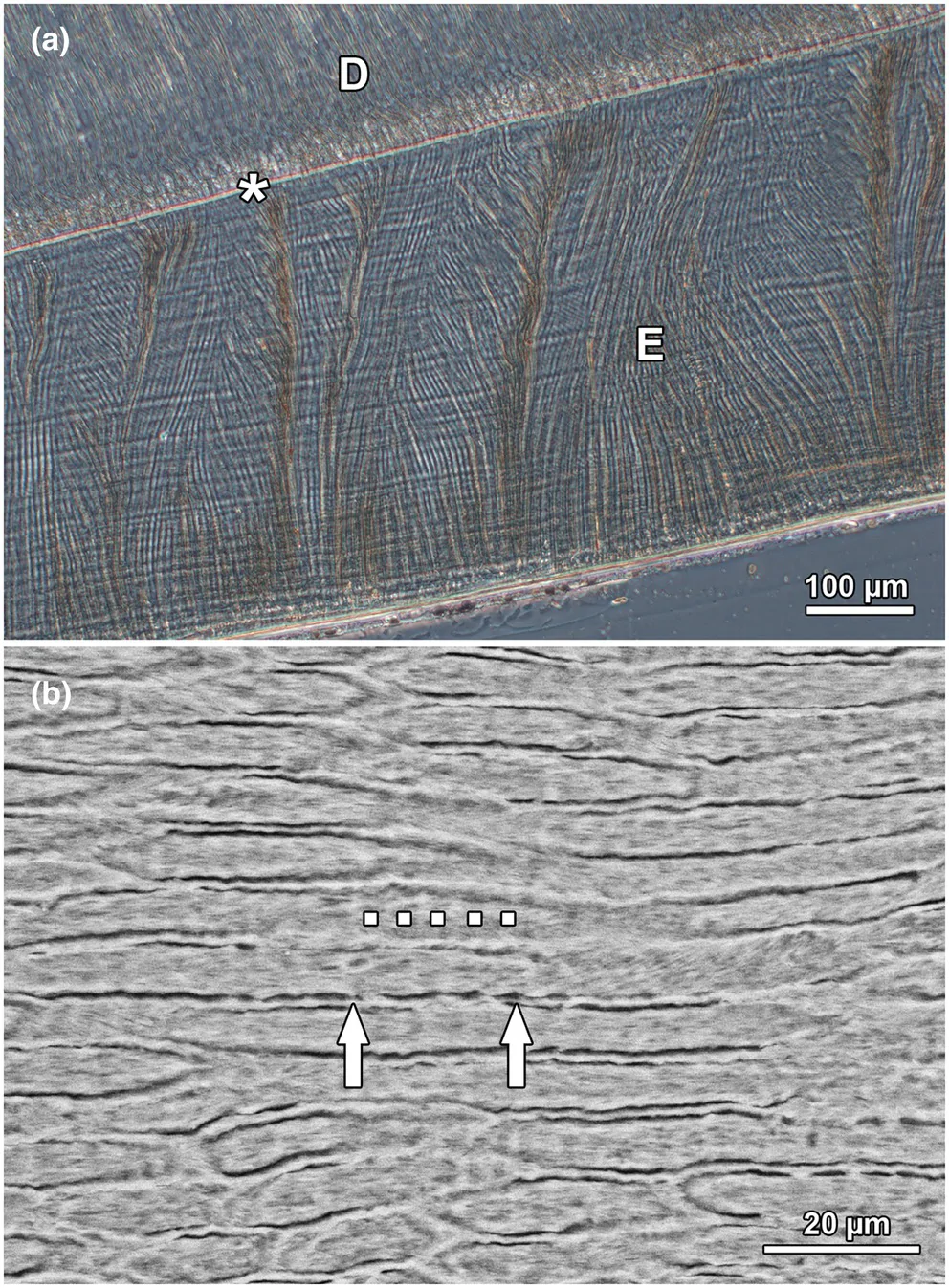
Enamel of the left M_1_ of a grey wolf (individual #1). (a) Micrograph of buccolingual ground section showing buccal enamel (E) of the cervical crown region. Section viewed in transmitted light with phase‐contrast enhancement, cuspal to the right. The dominant incremental markings are laminations that are steeply inclined and terminate at the outer enamel surface (OES) without forming perikyma grooves. Prominent Hunter–Schreger bands extend from the enamel–dentine junction (EDJ) (asterisk) through almost the entire enamel. D, dentine. (b) SEM‐backscattered electron (BSE) image of polished and etched sectional block surface, showing outer enamel of the mid‐lateral buccal crown region, cuspal to the right. Five subdaily growth increments (white squares) between two consecutive laminations (arrows) are indicated. The width of the daily growth increment between the marked laminations is approximately 20 μm.

**FIGURE 9:**
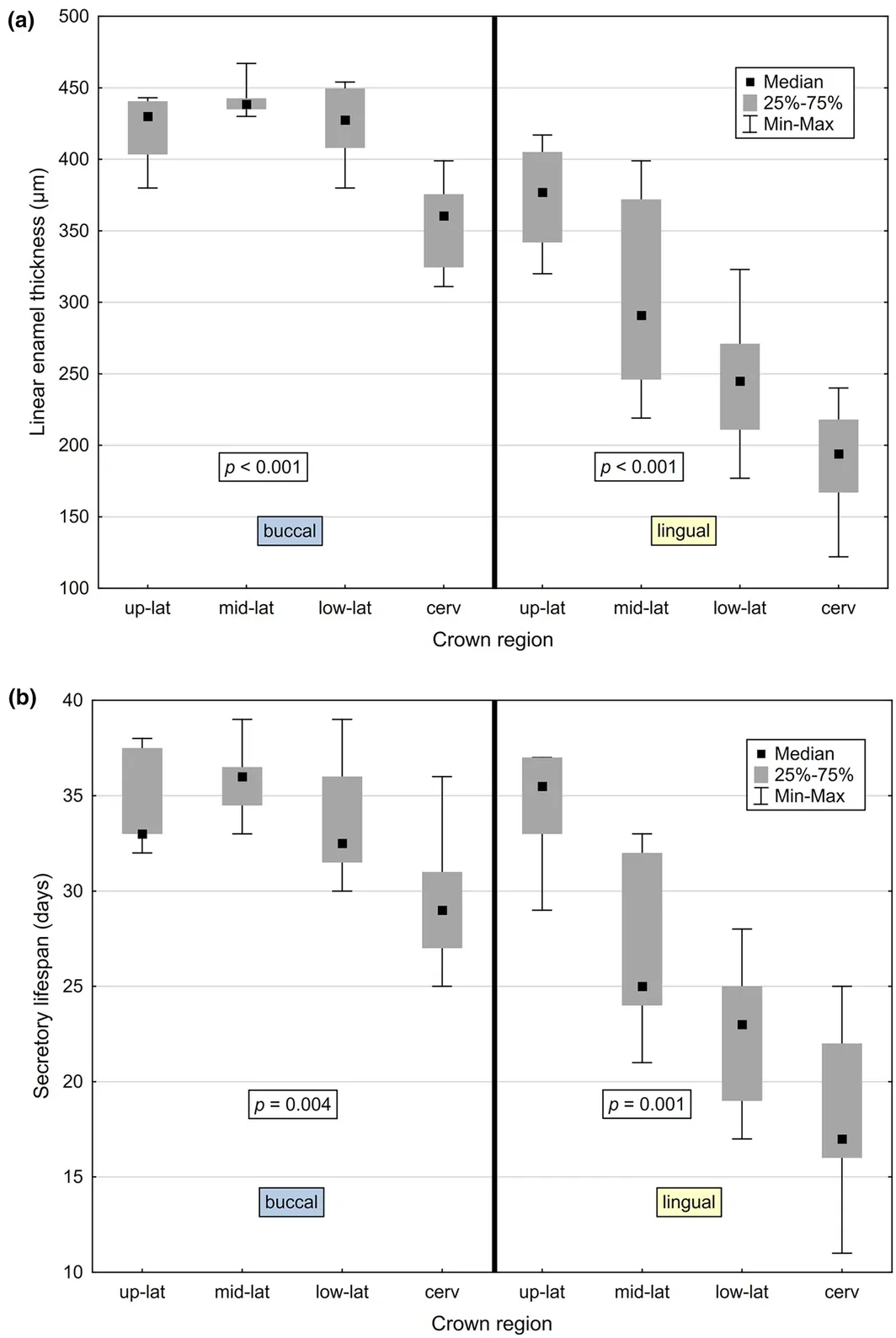
(a) Linear enamel thickness (LET, μm) and (b) ameloblast secretory lifespan (ASL, days) in the four distinguished crown regions (up‐lat = upper lateral, mid‐lat = mid‐lateral, low‐lat = lower lateral, cerv = cervical) of the buccal and lingual crown side of the left M_1_ of the red foxes. *p*‐values for the differences among the four crown regions for the buccal and lingual sides are given (Friedman ANOVA by ranks).

**FIGURE 10:**
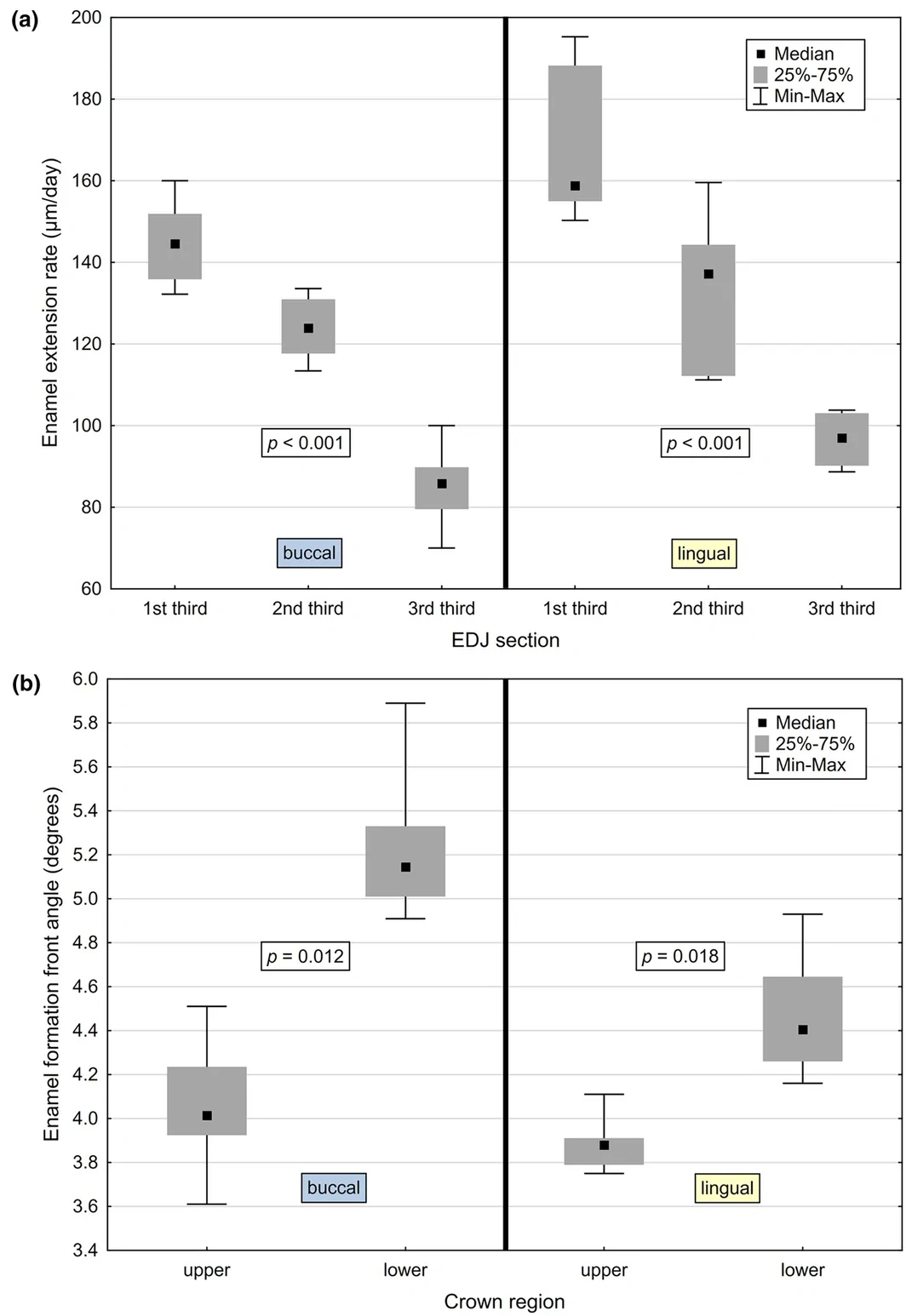
(a) Enamel extension rate (EER, μm/day) of the 1st (cuspal), 2nd (middle) and 3rd (cervical) third of the enamel–dentine junction (EDJ) length in buccal and lingual enamel of the red fox left M_1_. *p*‐values for the differences among the three EDJ sections for the buccal and lingual sides are given (Friedman ANOVA by ranks). (b) Enamel formation front angle (EFFa, degrees) for upper and lower crown portions in buccal and lingual enamel of the red fox left M_1_. *p*‐values for the difference between the two crown portions are given (Wilcoxon matched pairs test).

Corresponding to the situation in the red fox molars, also in the enamel of the grey wolf M_1_, the most prominent incremental markings were laminations that likewise followed a steeply inclined course from the EDJ to the OES (Figure 8a). Lowest DSR values in the range of 6–7 μm/day occurred close to the EDJ, while highest values (~20 μm/day) were recorded in outer buccal enamel (Figure 8b). Five subdaily growth increments were discernible between consecutive laminations (Figure 8b). Like in the red fox M_1_s, a NNL was not discernible in the enamel of the wolf M_1_.

### 3.2 LET and ASL

LET of the red fox M_1_ varied both between the buccal and lingual crown sides as well as along the cuspal‐cervical tooth axis (Figures 2 and 9a; Table S3). The highest mean value (441.1 μm) was recorded for buccal mid‐lateral enamel, the lowest mean value (190.7 μm) in lingual cervical enamel. Buccally, LET varied significantly among the four distinguished crown regions (Friedman ANOVA by ranks, *p* < 0.001) but was not significantly different (Friedman ANOVA by ranks, *p* = 0.197) when only the three lateral crown regions were compared. By contrast, on the lingual side, all four crown regions (Friedman ANOVA by ranks, *p* < 0.001) as well as the three lateral crown regions (Friedman ANOVA by ranks, *p* = 0.002) varied significantly in LET. The results of the pairwise post‐hoc comparisons of the four crown regions are given in Table S4.

The results for the secretory lifespan of the ameloblasts (ASL) mirrored those for LET (Figure 9b; Tables S3 and S5), with significant differences among the four crown regions (Friedman ANOVA by ranks, buccal: *p* = 0.004, lingual: *p* = 0.001). The results of the pairwise post‐hoc comparisons of the four crown regions are given in Table S5. If only the three lateral crown regions were compared, there was a significant difference for the lingual (Friedman ANOVA by ranks, *p* = 0.006) but not the buccal side (Friedman ANOVA by ranks, *p* = 0.150). Comparison between buccal and lingual tooth sides showed no significant difference in ASL for the upper lateral crown region (Wilcoxon matched pairs test, *p* = 0.273), whereas for the other three regions, the side differences were significant (Wilcoxon matched pairs tests, all *p*‐values <0.05), with the values for buccal exceeding those for lingual enamel.

LET in the grey wolf M_1_ was higher compared with corresponding crown regions of the red fox M_1_. Maximum values of about 750 μm were reached in the upper lateral and mid‐lateral buccal crown regions of the wolf M_1_.

### 3.3 EER and EFFa

The recorded EERs for the respective thirds of the EDJ length of the buccal and lingual crown sides of the red fox M_1_s are indicated in Figure 10a and given in Table S6. Values were highest in the first (cuspal) third (mean values of 144.6 μm/day in buccal and of 170.3 μm/day in lingual enamel) and lowest in the cervical third (mean values of 85.0 μm/day in buccal and of 96.7 μm/day in lingual enamel). The differences among the three sections were significant (Friedman ANOVA by ranks, *p* < 0.001) for both crown sides. Pairwise post‐hoc comparisons between the thirds revealed significant differences for buccal enamel (all nominal *p*‐values = 0.012, adjusted *p*‐values = 0.036), while lingually the differences were just outside the limit of statistical significance following Bonferroni correction (all nominal *p*‐values = 0.018, adjusted *p*‐values = 0.054). Values for corresponding EDJ sections were significantly higher on the lingual compared with the buccal crown side for the 1st (cuspal) and 3rd (cervical) thirds (Wilcoxon matched pairs tests, *p* = 0.018) but did not vary significantly for the 2nd (middle) third (Wilcoxon matched pairs test, *p* = 0.237).

Mean EFFa values were significantly lower in upper compared with lower crown portions of the respective crown side (buccal upper: 4.1°, buccal lower: 5.2°, Wilcoxon matched pairs test, *p* = 0.012; lingual upper: 3.9°, lingual lower: 4.5°, Wilcoxon matched pairs test, *p* = 0.018) (Figure 10b; Table S6). The side difference between corresponding crown portions was significant for the lower location (Wilcoxon matched pairs test, *p* = 0.012) but not the upper one (Wilcoxon matched pairs test, *p* = 0.063).

### 3.4 Prism decussation

The enamel of both red fox and grey wolf exhibited a pronounced enamel prism decussation throughout most of its thickness. This caused the presence of prominent Hunter–Schreger bands with alternating diazones (exhibiting more or less cross‐sectioned prisms) and parazones (exhibiting tangentially cut prisms). A small surface zone showed radial enamel (Figures 6a,b and 8a).

## 4 DISCUSSION

### 4.1 Characterization of incremental markings and calculation of CFT

The present study for the first time provides a detailed histological analysis of incremental markings in red fox enamel. Based on the counts of daily laminations, an average CFT of 102 days (range 99–107 days) was reconstructed for the M_1_ of this species.

In farmed red foxes, emergence of the M_1_ protoconid tip through the gum was reported at the earliest at postnatal day 105 and consistently at 112 days of age (Gauß, 1939). In hand‐reared red foxes, Linhart (1968) observed the emergence of the M_1_ through the gum between postnatal days 112 and 119. The reconstructed mean CFT of about 102 days for this tooth fits well with these data, as a few days of root growth must be added after crown completion prior to tooth eruption. Comparison with the results of the above studies clearly supports the identification of laminations in canid enamel as daily incremental markings.

In domestic dogs, the first molar is the only tooth of the permanent dentition that starts mineralization prior to the end of the gestation period of ~60 days. Initial mineralization of the protoconid tip of the M_1_ occurs at gestation day 55 (Williams & Evans, 1978). The CFT for the red fox M_1_ calculated by us in combination with the data on its eruption indicates that crown mineralization of this tooth does either start postnatally or immediately prior to the end of the 51–53 days gestation period (Wandeler & Lüps, 1993; Yatu et al., 2019). This conclusion is supported by the lack of a NNL in the enamel of all studied red fox M_1_s. Early postnatal onset of crown formation in the M_1_ has also been observed in other carnivoran species. Thus, Berkovitz (1973) and Popowics (1998) report the start of M_1_ crown formation in *Mustela putorius* (Mustelidae) at postnatal day 3. Likewise, Dittrich (1960) observed onset of M_1_ crown formation in the brown bear, *Ursus arctos* (Ursidae), at a postnatal age of 3 days.

Based on our findings, it is concluded that previous characterizations of enamel incremental markings in canid teeth (Fukuhara, 1959; Sazelova et al., 2020) are incorrect as a consequence of misinterpretation of daily as supradaily markings (long‐period striae of Retzius) and of subdaily markings as daily prism cross‐striations. Due to this error, the previously reported DSR values of 2.4 μm/day for dog enamel (Fukuhara, 1959) and of 4.1 μm/day for grey wolf enamel (Sazelova et al., 2020) are far too low. As a consequence, the CFT of about a year (361 days) calculated for a wolf P^4^ by Sazelova et al. (2020) is much too long. This is also obvious when considering that the permanent dentition of the wolf is in occlusion at about 5–5.5 months of postnatal age (Geiger et al., 2016; Peters, 1993) and that the fourth permanent premolar in dogs starts crown mineralization only after birth (Williams & Evans, 1978). Actually, the CFT for the P^4^ of wolves and dogs, forming the maxillary part of the carnassial complex, should be similar to that of the red fox M_1_ analyzed in the present study. Rapid crown formation has also been observed in the brown bear M_1_, with eruption through the gum occurring between postnatal days 127 and 154 (Dittrich, 1960).

Although they provide no data for dental growth parameters in their study, the characterization of incremental markings in enamel and dentine of permanent canine teeth from various domestic dog breeds given by Hogg et al. (2018) apparently suffers from the same misidentification of incremental markings as the work by Fukuhara (1959) and Sazelova et al. (2020). Hogg et al. (2018) report a Retzius periodicity (repeat interval) between four and 6 days based on the analysis of incremental markings diagnosed by them as daily prism cross‐striations (see figure 2 in Hogg et al., 2018) between (presumed) striae of Retzius. However, if this interpretation were correct, the resulting DSR would only be about 5 μm/day. This value is below the minimum DSR recorded in our study for enamel adjacent to the EDJ in the red fox M_1_. Applying this low DSR to the crown of the studied dog canines would lead to unrealistically long CFTs, as the permanent canines of dogs are fully erupted at about 5–6 months of age (Habermehl, 1975). An alternative interpretation of the histological structures depicted by Hogg et al. (2018) is that the cross‐striations do not represent daily but subdaily growth increments and that the alleged supradaily striae of Retzius are actually daily laminations. In the present study, four to six subdaily growth increments were recorded between consecutive laminations in red fox and grey wolf enamel. For the M_1_ of the latter species, we recorded a DSR of about 20 μm in outer buccal enamel, a value that is four times that reported for dog enamel by Hogg et al. (2018). However, if the interpretation of the enamel incremental markings is changed as suggested by us, the enamel growth rate in dog canines would be in overall agreement with those obtained for red fox and grey wolf molars by us.

Based on the above discussion, we conclude that previous studies on canid enamel suffered from the same misidentification of incremental markings that has also occurred in previous studies of bovid, suid, and equid enamel, as detailed by Kierdorf et al. (2013, 2014, 2019) and Nacarino‐Meneses et al. (2017). An important factor provoking such misidentification is the much higher rate of enamel formation in ungulate and canid teeth compared to that of primates, especially humans. In the latter, the DSR ranges between 2 and 3 μm near the EDJ and 6 and 7 μm in outer enamel (Smith, 2006). By contrast, in the enamel of ungulates (Cuccu et al., 2025, 2026; Emken et al., 2023; Hullot et al., 2026; Kierdorf et al., 2013, 2014, 2019; Nacarino‐Meneses et al., 2017, 2025; Nacarino‐Meneses & Chinsamy, 2022) and canids (present study), a DSR of 6–7 μm is near the lower limit of the range, while maxima approach or even exceed 20 μm/day. A second factor is the similarity in microscopic appearance between daily (laminations) and subdaily (subdaily prism cross‐striations) incremental markings in ungulate and canid enamel and, respectively, long‐period (striae of Retzius) and daily incremental markings (daily prism cross‐striations) in primate enamel (Emken et al., 2021; Hullot et al., 2026; Kierdorf et al., 2013, 2014, 2019).

### 4.2 Comparison of enamel growth parameters in canids and other mammalian taxa

The DSR values of the red fox M_1_s showed a characteristic variation among the distinguished enamel zones, with lowest apposition rates in the inner and highest in the outer third of the enamel layer. A similar trend of increasing DSR from inner to outer enamel was previously also found in lateral enamel of goat and sheep M_1_ (Kierdorf et al., 2012, 2013), in M_2_ and M_3_ of wild boar and domestic pigs (Emken et al., 2023; Kierdorf et al., 2014, 2019), and M_1_ and M_3_ of giraffes (Nacarino‐Meneses et al., 2025). However, the differences in DSR between the enamel zones of the giraffe molars were less pronounced than those found in the pig and red fox molars. For permanent premolars and molars of different hominid species, Beynon et al. (1991) have previously also reported a trend of increasing DSR from inner to outer enamel, however, at overall much lower secretion rates. Contrary to the above findings, in molars of extant equids (Nacarino‐Meneses et al., 2017) and fossil notoungulates (Hullot et al., 2026), no significant variations of DSR were recorded between inner and outer enamel. Whether this discrepancy reflects a sampling effect or points to species differences with respect to this growth parameter remains to be elucidated.

In the red fox M_1_, the DSRs recorded in the inner enamel zones were similar for buccal and lingual enamel, while in central and outer enamel, the DSR was higher buccally. Highest mean DSR values (17.3 and 17.4 μm/day, respectively) were recorded in outer enamel of the buccal upper lateral and mid‐lateral crown zones, while the respective values for the corresponding lingual crown zones were only 12.7 and 12.5 μm/day. The DSR for buccal outer enamel of red fox M_1_s is in the same range as the highest mean DSRs previously reported for different ungulate taxa, including cervids (Cuccu et al., 2025, 2026; Iinuma et al., 2004), giraffids (Nacarino‐Meneses et al., 2025), bovids (Cuccu et al., 2026; Jordana et al., 2014; Kierdorf et al., 2013), equids (Nacarino‐Meneses et al., 2017; Orlandi‐Oliveras et al., 2019), and toxodont notoungulates (Hullot et al., 2026). Even higher mean DSRs (between 23 and 26.5 μm/day) have been recorded in outer enamel of mandibular second molars from domestic pigs and wild boar that exhibit very thick enamel (Emken et al., 2023). By contrast, much lower values were reported for molars of elephantids (DSR of 2–5 μm; Dirks et al., 2012) and two archaic ungulates, *Meniscotherium* and *Phenacodus* (~2.5–5 μm; Dirks et al., 2009). These low values may reflect very low enamel growth rates in these taxa; however, it cannot be ruled out that they are also the result of the misinterpretation of enamel incremental growth marks discussed above, as was previously also assumed by Nacarino‐Meneses et al. (2025).

The differences in DSR between buccal and lingual enamel of corresponding crown regions of the red fox M_1_ are related to differences in LET. These were especially pronounced in the mid‐lateral, lower lateral, and cervical crown regions, with the values for lingual enamel being, respectively, only about 69%, 58%, and 54% of those for buccal enamel. Also, the duration of appositional activity by the ameloblasts (ASL) was lower lingually. By contrast, side differences in LET were less pronounced in the upper lateral crown zone, where reconstructed ASLs were similar for buccal and lingual enamel. A close relationship between LET and ASL was previously demonstrated also in porcine molars, which in the lateral crown portions of the wild boar M_3_ exhibit mean LET values of more than 2000 μm and average ASLs of about 5 months (Kierdorf et al., 2019).

The EER in the red fox M_1_s decreased from the upper third to the lower third of the EDJ length, with mean and median values for the lingual crown side always exceeding those for the corresponding region on the buccal side. The decrease in EER from the upper to the lower third of the buccal and lingual EDJ length was moderate. Thus, buccally the lower third still showed ~59% of the EER recorded for the upper third, while lingually the corresponding value was ~57%. In other species, a more marked decrease in EER from the upper to the lower crown third has been observed, the latter exhibiting less than 20% of the value recorded for the former (wild boar M_2_: buccal ~19%; lingual ~12%, Emken et al., 2023; sheep M_1_: buccal ~19%, Kierdorf et al., 2013; *Anchitherium* M_1_: buccal ~17%, Calderon et al., 2026). It has been suggested that the EER is the main factor responsible for a rapid increase of crown height and a short CFT (Dirks et al., 2012; Emken et al., 2023; Hullot et al., 2026).

The mean EFFa in the upper crown portion of the red fox M_1_s was very small (4.1° buccally and 3.9° lingually) and only slightly increased in the lower crown portion (5.2° buccally and 4.5° lingually). Thus, both the EER and the EFFa indicate a very rapid crown elongation in the red fox M_1_, which is also reflected by the very steep inclination of the laminations throughout the enamel. In consequence, the laminations reach the OES at very small angles. It has previously been suggested (Jordana et al., 2014) that EEFa values positively correlate with certain life history variables, with narrow angles indicating more rapid life history traits, including an early age at first reproduction in ruminants. However, this has been questioned by other authors (Nacarino‐Meneses et al., 2025) who argue that the relationship between EEFa and life history traits is more complex since the former also has an important phylogenetic background. While this caveat is undisputed, the findings in the red fox, a species that achieves sexual maturity already at 9–12 months of age (Wandeler & Lüps, 1993), do at least not contradict the statement by Jordana et al. (2014). Our results show that the speed of crown elongation, not the DSR, is the main factor enabling the rapid formation of the relatively large M_1_ of the red fox in only about 3.5 months. The fast dental development of canids is considered essential for their very rapid postnatal development, with weaning occurring already about 4–6 weeks after birth in red foxes and grey wolves (Peters, 1993; Wandeler & Lüps, 1993).

## 5 CONCLUSIONS

Our histological analysis identified laminations as the most prominent incremental markings in the enamel of the red fox and grey wolf. The reconstructed CFT of the red fox M_1_ fits the known eruption time for this tooth, thereby indicating a daily periodicity of these incremental markings. Four to six subdaily growth increments were observed between consecutive laminations, while supradaily incremental markings (striae of Retzius) were not discernible. The established growth parameters (CFT, DSR, EER, EFFa) of the red fox M_1_ demonstrate a rapid progression of crown elongation, enabling the formation of a relatively large tooth crown in less than 4 months.

The findings of the present investigation indicate that previous studies of canid teeth (Fukuhara, 1959; Hogg et al., 2018; Sazelova et al., 2020) misidentified the enamel incremental markings. Daily laminations were mistaken for supradaily striae of Retzius and subdaily prism cross‐striations for daily cross‐striations. As a consequence, these studies reported too low DSRs and too high CFTs for the canid teeth. As has been previously stated (Kierdorf et al., 2013, 2014, 2019), the interpretive scheme initially developed for the characterization of incremental markings in (slowly forming) primate enamel must not uncritically be extended to other mammalian orders with much higher enamel growth rates. Clearly, the slow formation rate of primate (especially hominid) enamel constitutes the exception, not the rule.

Recently, Hullot et al. (2026) proposed a methodological framework for analyzing growth parameters of ungulate enamel. The results of the present study demonstrate that this methodological framework can also be applied to canid enamel. As has been repeatedly stressed (Calderon et al., 2026; Hogg, 2018; Hullot et al., 2026; Nacarino‐Meneses et al., 2025), studying enamel growth parameters in extant species, for which life history data are available, enables checking for the correct interpretation of histologically determined periodicities of dental growth marks. Transferring such calibrated interpretive schemes to related extinct species, for which life history data are not available, allows a reliable assessment of dental growth parameters also in these species.

It remains to be explored whether our findings on canid enamel can be extended to other carnivoran families. Further studies are necessary to broaden our knowledge on enamel incremental structures in this order as well as in other mammalian taxa.

## AUTHOR CONTRIBUTIONS

H.K. and U.K. conceived and designed the study; H.K. performed the histological analysis and wrote the first draft; H.K. and U.K. analyzed the data, prepared the figures and tables, and revised the manuscript.

## CONFLICT OF INTEREST STATEMENT

The authors declare no conflicts of interest.

## Supporting information


**Table S1.** Enamel daily secretion rate (DSR, μm/day) in different crown regions of red fox left M_1_. bu, buccal; li, lingual; *n1*, number of teeth for which data were obtained; *n2*, total number of measurements. When more than one measurement per tooth was performed at a given position, the mean value from these measurements was used for further calculation.
**Table S2.** Significance of differences in enamel daily secretion rate (DSR) among different enamel areas (inner, central, and outer third) of buccal and lingual crown regions of red fox M_1_. *p*‐values <0.05 are given in bold.
**Table S3.** Linear enamel thickness (LET, μm) and ameloblast secretory lifespan (ASL, days) for different crown regions of red fox left M_1_. bu, buccal; li, lingual; *n*, number of teeth for which data were obtained.
**Table S4.** Significance of differences in linear enamel thickness (LET) among the four different crown regions (upper lateral, mid‐lateral, lower lateral, cervical) of red fox M_1_. *p*‐values <0.05 are given in bold.
**Table S5.** Significance of differences in ameloblast secretory lifespan (ASL) among the four different crown regions (upper lateral, mid‐lateral, lower lateral, cervical) of red fox M_1_. *p*‐values <0.05 are given in bold.
**Table S6.** Enamel extension rate (EER, μm/day) and enamel formation front angle (EFFa, degrees) in the buccal and lingual crown portions of red fox left M_1_. bu, buccal; li, lingual; *n*, number of teeth for which data were obtained.

## Data Availability

The data that support the findings of the study are available from the corresponding author upon reasonable request.
