# Supplementary material for: Histological analysis of incremental markings and crown growth characteristics in mandibular first molars of the red fox, Vulpes vulpes (Canidae, Mammalia)
Source: J Anat. 2026 May 18;249(6):1445–60. doi: 10.1111/joa.70178 (PMC13399015; doi:10.1111/joa.70178)
Supplement: Supplementary file 1 [file JOA-249-1445-s001.docx]

**SUPPLEMENTARY INFORMATION**

TABLE S1

Enamel daily secretion rate (DSR, µm/day) in different crown regions of red fox left M_1_, bu – buccal, li – lingual, *n1* – number of teeth for which data were obtained, *n2* – total number of measurements. When more than one measurement per tooth was performed at a given position, the mean value from these measurements was used for further calculation.

| Crown region | *n1/n2* | Mean | SD | Median | Minimum | Maximum |
| --- | --- | --- | --- | --- | --- | --- |
| bu, inner, upper lateral | 6/11 | 7.98 | 0.63 | 7.80 | 7.30 | 8.80 |
| bu, inner, mid-lateral | 8/16 | 7.93 | 0.96 | 8.05 | 6.60 | 9.60 |
| bu, inner, lower lateral | 8/17 | 7.69 | 0.57 | 7.80 | 6.50 | 8.40 |
| bu, inner, cervical | 8/13 | 7.38 | 0.66 | 7.40 | 6.20 | 8.30 |
| bu, central, upper-lateral | 8/18 | 12.25 | 1.10 | 12.55 | 10.00 | 13.20 |
| bu, central, mid-lateral | 8/17 | 13.11 | 1.04 | 12.80 | 12.00 | 15.10 |
| bu, central, lower lateral | 8/18 | 12.53 | 0.90 | 12.20 | 11.50 | 14.00 |
| bu, central, cervical | 8/14 | 12.54 | 1.40 | 12.65 | 10.90 | 15.10 |
| bu, outer, upper lateral | 8/36 | 17.33 | 1.34 | 17.05 | 15.80 | 19.20 |
| bu, outer, mid-lateral | 8/34 | 17.35 | 0.92 | 17.45 | 16.10 | 18.60 |
| bu, outer, lower lateral | 8/34 | 16.69 | 1.09 | 16.90 | 14.50 | 18.30 |
| bu, outer, cervical | 8/22 | 16.04 | 2.20 | 16.90 | 12.50 | 17.80 |
| li, inner, upper lateral | 5/7 | 7.84 | 0.48 | 7.80 | 7.30 | 8.60 |
| li, inner, mid-lateral | 7/9 | 7.76 | 0.50 | 7.90 | 6.90 | 8.40 |
| li, inner, lower lateral | 7/9 | 7.63 | 0.92 | 7.80 | 6.00 | 8.60 |
| li, inner, cervical | 7/7 | 6.81 | 0.95 | 6.70 | 5.30 | 8.10 |
| li, central, upper lateral | 7/13 | 10.93 | 0.60 | 11.00 | 10.00 | 11.80 |
| li, central, mid-lateral | 7/13 | 10.00 | 1.27 | 10.30 | 7.70 | 11.40 |
| li, central, lower lateral | 7/11 | 10.14 | 1.07 | 10.10 | 8.20 | 11.20 |
| li, central, cervical | 6/6 | 9.42 | 0.37 | 9.50 | 8.80 | 9.80 |
| li, outer, upper lateral | 7/11 | 12.67 | 0.96 | 12.80 | 11.00 | 13.70 |
| li, outer, mid-lateral | 7/12 | 12.51 | 1.36 | 12.10 | 10.50 | 14.60 |
| li, outer, lower lateral | 7/13 | 11.91 | 1.48 | 12.30 | 9.00 | 13.10 |
| li, outer, cervical | 7/10 | 11.07 | 1.63 | 11.00 | 8.40 | 13.10 |

TABLE S2

Significance of differences in enamel daily secretion rate (DSR) among different enamel areas (inner, central and outer third) of buccal and lingual crown regions of red fox M_1_. *P*-values < 0.05 are given in bold.

| Crown side | Crown region | Significance of differences among enamel areas (inner, central, outer third)^1^ | Pairwise comparisons^2^ | Nominal *p*-value/adjusted *p*-value^3^ |
| --- | --- | --- | --- | --- |
| buccal | upper lateral | ***p* = 0.002** | inner *vs* central | **0.028**/0.084 |
|  |  |  | inner *vs* outer | **0.028**/0.084 |
|  |  |  | central *vs* outer | **0.012/0.036** |
|  | midlateral | ***p* < 0.001** | inner *vs* central | **0.012/0.036** |
|  |  |  | inner *vs* outer | **0.012/0.036** |
|  |  |  | central *vs* outer | **0.012/0.036** |
|  | lower lateral | ***p* < 0.001** | inner *vs* central | **0.012/0.036** |
|  |  |  | inner *vs* outer | **0.012/0.036** |
|  |  |  | central *vs* outer | **0.012/0.036** |
|  | cervical | ***p* < 0.001** | inner *vs* central | **0.012/0.036** |
|  |  |  | inner *vs* outer | **0.012/0.036** |
|  |  |  | central *vs* outer | **0.012/0.036** |
| lingual | upper lateral | ***p* = 0.007** | inner *vs* central | **0.043**/0.129 |
|  |  |  | inner *vs* outer | **0.043**/0.129 |
|  |  |  | central *vs* outer | **0.018**/0.054 |
|  | midlateral | ***p* < 0.001** | inner *vs* central | **0.018**/0.054 |
|  |  |  | inner *vs* outer | **0.018**/0.054 |
|  |  |  | central *vs* outer | **0.018**/0.054 |
|  | lower lateral | ***p* < 0.001** | inner *vs* central | **0.018**/0.054 |
|  |  |  | inner *vs* outer | **0.018**/0.054 |
|  |  |  | central *vs* outer | **0.018**/0.054 |
|  | cervical | ***p* = 0.002** | inner *vs* central | **0.028**/0.084 |
|  |  |  | inner *vs* outer | **0.018**/0.054 |
|  |  |  | central *vs* outer | **0.028**/0.084 |

^1^Friedman ANOVA by ranks

^2^Wilcoxon matched pairs test

^3^Bonferroni adjusted *p*-value

TABLE S3

Linear enamel thickness (LET, µm) and ameloblast secretory lifespan (ASL, days) for different crown regions of red fox left M_1_, bu – buccal, li – lingual, *n* – number of teeth for which data were obtained.

| Variable/crown region | *n* | Mean | SD | Median | Minimum | Maximum |
| --- | --- | --- | --- | --- | --- | --- |
| LET, bu, upper lateral | 8 | 421.4 | 23.2 | 430.0 | 380.0 | 443.0 |
| LET, bu, mid-lateral | 8 | 441.1 | 11.3 | 438.5 | 430.0 | 467.0 |
| LET, bu, lower lateral | 8 | 425.5 | 26.0 | 427.5 | 380.0 | 454.0 |
| LET, bu, cervical | 8 | 353.9 | 31.0 | 360.5 | 311.0 | 399.0 |
| LET, li, upper lateral | 7 | 374.0 | 34.0 | 377.0 | 320.0 | 417.0 |
| LET, li, mid-lateral | 7 | 304.0 | 64.6 | 291.0 | 219.0 | 399.0 |
| LET, li, lower lateral | 7 | 247.7 | 46.8 | 245.0 | 177.0 | 323.0 |
| LET, li, cervical | 7 | 190.7 | 39.3 | 194.0 | 122.0 | 240.0 |
| ASL, bu, upper lateral | 8 | 34.6 | 2.6 | 33.0 | 32.0 | 38.0 |
| ASL, bu, mid-lateral | 8 | 35.8 | 2.0 | 36.0 | 33.0 | 39.0 |
| ASL, bu, lower lateral | 8 | 33.6 | 3.5 | 32.5 | 30.0 | 39.0 |
| ASL, bu, cervical | 8 | 29.4 | 3.4 | 29.0 | 25.0 | 36.0 |
| ASL, li, upper lateral | 6 | 34.5 | 3.2 | 35.5 | 29.0 | 37.0 |
| ASL, li, mid-lateral | 7 | 26.9 | 4.4 | 25.0 | 21.0 | 33.0 |
| ASL, li, lower lateral | 7 | 22.6 | 3.7 | 23.0 | 17.0 | 28.0 |
| ASL, li, cervical | 7 | 18.1 | 4.5 | 17.0 | 11.0 | 25.0 |

TABLE S4

Significance of differences in linear enamel thickness (LET) among the four different crown regions (upper lateral, mid-lateral, lower lateral, cervical) of red fox M_1_. *P*-values < 0.05 are given in bold.

| Crown side | Significance of differences among the 4 crown regions^1^ | Pairwise comparisons^2^ | Nominal *p*-value/adjusted *p*-value^3^ |
| --- | --- | --- | --- |
| buccal | ***p* < 0.001** | upper lateral *vs* mid-lateral | 0.059/0.354 |
|  |  | upper lateral *vs* lower lateral | 0.674/1 |
|  |  | upper lateral *vs* cervical | **0.012**/0.072 |
|  |  | mid-lateral *vs* lower lateral | 0.107/0.642 |
|  |  | mid-lateral *vs* cervical | **0.012**/0.072 |
|  |  | lower lateral *vs* cervical | **0.012**/0.072 |
| lingual | ***p* < 0.001** | upper lateral *vs* mid-lateral | **0.018**/0.108 |
|  |  | upper lateral *vs* lower lateral | **0.018**/0.108 |
|  |  | upper lateral *vs* cervical | **0.018**/0.108 |
|  |  | mid-lateral *vs* lower lateral | **0.018**/0.108 |
|  |  | mid-lateral *vs* cervical | **0.018**/0.108 |
|  |  | lower lateral *vs* cervical | **0.018**/0.108 |

^1^Friedman ANOVA by ranks

^2^Wilcoxon matched pairs test

^3^Bonferroni adjusted *p*-value

TABLE S5

Significance of differences in ameloblast secretory lifespan (ASL) among the four different crown regions (upper lateral, mid-lateral, lower lateral, cervical) of red fox M_1_. *P*-values < 0.05 are given in bold.

| Crown side | Significance of differences among the 4 crown regions^1^ | Pairwise comparisons^2^ | Nominal *p*-value/adjusted *p*-value^3^ |
| --- | --- | --- | --- |
| buccal | ***p* = 0.004** | upper lateral *vs* mid-lateral | **0.012**/0.072 |
|  |  | upper lateral *vs* lower lateral | **0.012**/0.072 |
|  |  | upper lateral *vs* cervical | **0.012**/0.072 |
|  |  | mid-lateral *vs* lower lateral | 0.076/0.456 |
|  |  | mid-lateral *vs* cervical | **0.018**/0.108 |
|  |  | lower lateral *vs* cervical | **0.012**/0.072 |
| lingual | ***p* = 0.001** | upper lateral *vs* mid-lateral | **0.028**/0.168 |
|  |  | upper lateral *vs* lower lateral | **0.028**/0.168 |
|  |  | upper lateral *vs* cervical | **0.028**/0.168 |
|  |  | mid-lateral *vs* lower lateral | 0.052/0.312 |
|  |  | mid-lateral *vs* cervical | **0.028**/0.168 |
|  |  | lower lateral *vs* cervical | **0.018**/0.108 |

^1^Friedman ANOVA by ranks

^2^Wilcoxon matched pairs test

^3^Bonferroni adjusted *p*-value

TABLE S6

Enamel extension rate (EER, µm/day) and enamel formation front angle (EFFa, degrees) in the buccal and lingual crown portions of red fox left M_1_, bu – buccal, li – lingual, *n* – number of teeth for which data were obtained.

| Crown region | *n* | Mean | SD | Median | Minimum | Maximum |
| --- | --- | --- | --- | --- | --- | --- |
| EER, bu, 1st (cuspal) third | 8 | 144.6 | 9.9 | 144.6 | 132.2 | 160.0 |
| EER, bu, 2nd (middle) third | 8 | 124.0 | 7.8 | 124.0 | 113.4 | 133.6 |
| EER, bu, 3rd (cervical) third | 8 | 85.0 | 9.1 | 85.8 | 70.0 | 100.0 |
| EER, li, 1st (cuspal) third | 7 | 170.3 | 18.7 | 158.8 | 150.3 | 195.3 |
| EER, li, 2nd (middle) third | 7 | 132.7 | 17.8 | 137.2 | 111.2 | 159.6 |
| EER, li, 3rd (cervical) third | 7 | 96.7 | 6.3 | 97.0 | 88.7 | 103.8 |
| EFFa, bu, upper | 8 | 4.06 | 0.28 | 4.02 | 3.61 | 4.51 |
| EFFa, bu, lower | 8 | 5.22 | 0.32 | 5.15 | 4.91 | 5.89 |
| EFFa, li, upper | 7 | 3.88 | 0.12 | 3.88 | 3.75 | 4.11 |
| EFFa, li, lower | 8 | 4.46 | 0.27 | 4.41 | 4.16 | 4.93 |
