# Supplementary material for: Histological analysis of incremental markings and crown growth characteristics in mandibular first molars of the red fox, Vulpes vulpes (Canidae, Mammalia)
Source: J Anat. 2026 May 18:10.1111/joa.70178. Online ahead of print. doi: 10.1111/joa.70178 (PMC13399015; doi:10.1111/joa.70178)
Supplement: Supplementary file 1 — Table S1. Enamel daily secretion rate (DSR, μm/day) in different crown regions of red fox left M1. bu, buccal; li, lingual; n1, number of teeth for which data were obtained; n2, total number of measurements. When more than one measurement per tooth was performed at a given position, the mean value from these measurements was used for further calculation. Table S2. Significance of differences in enamel daily secretion rate (DSR) among different enamel areas (inner, central, and outer third) of buccal and lingual crown regions of red fox M1. p‐values <0.05 are given in bold. Table S3. Linear enamel thickness (LET, μm) and ameloblast secretory lifespan (ASL, days) for different crown regions of red fox left M1. bu, buccal; li, lingual; n, number of teeth for which data were obtained. Table S4. Significance of differences in linear enamel thickness (LET) among the four different crown regions (upper lateral, mid‐lateral, lower lateral, cervical) of red fox M1. p‐values <0.05 are given in bold. Table S5. Significance of differences in ameloblast secretory lifespan (ASL) among the four different crown regions (upper lateral, mid‐lateral, lower lateral, cervical) of red fox M1. p‐values <0.05 are given in bold. Table S6. Enamel extension rate (EER, μm/day) and enamel formation front angle (EFFa, degrees) in the buccal and lingual crown portions of red fox left M1. bu, buccal; li, lingual; n, number of teeth for which data were obtained. [file JOA-9999-0-s001.docx]

**SUPPLEMENTARY INFORMATION**

TABLE S1

Enamel daily secretion rate (DSR, µm/day) in different crown regions of red fox left M_1_, bu – buccal, li – lingual, *n1* – number of teeth for which data were obtained, *n2* – total number of measurements. When more than one measurement per tooth was performed at a given position, the mean value from these measurements was used for further calculation.

| Crown region | *n1/n2* | Mean | SD | Median | Minimum | Maximum |
| --- | --- | --- | --- | --- | --- | --- |
| bu, inner, upper lateral | 6/11 | 7.98 | 0.63 | 7.80 | 7.30 | 8.80 |
| bu, inner, mid-lateral | 8/16 | 7.93 | 0.96 | 8.05 | 6.60 | 9.60 |
| bu, inner, lower lateral | 8/17 | 7.69 | 0.57 | 7.80 | 6.50 | 8.40 |
| bu, inner, cervical | 8/13 | 7.38 | 0.66 | 7.40 | 6.20 | 8.30 |
| bu, central, upper-lateral | 8/18 | 12.25 | 1.10 | 12.55 | 10.00 | 13.20 |
| bu, central, mid-lateral | 8/17 | 13.11 | 1.04 | 12.80 | 12.00 | 15.10 |
| bu, central, lower lateral | 8/18 | 12.53 | 0.90 | 12.20 | 11.50 | 14.00 |
| bu, central, cervical | 8/14 | 12.54 | 1.40 | 12.65 | 10.90 | 15.10 |
| bu, outer, upper lateral | 8/36 | 17.33 | 1.34 | 17.05 | 15.80 | 19.20 |
| bu, outer, mid-lateral | 8/34 | 17.35 | 0.92 | 17.45 | 16.10 | 18.60 |
| bu, outer, lower lateral | 8/34 | 16.69 | 1.09 | 16.90 | 14.50 | 18.30 |
| bu, outer, cervical | 8/22 | 16.04 | 2.20 | 16.90 | 12.50 | 17.80 |
| li, inner, upper lateral | 5/7 | 7.84 | 0.48 | 7.80 | 7.30 | 8.60 |
| li, inner, mid-lateral | 7/9 | 7.76 | 0.50 | 7.90 | 6.90 | 8.40 |
| li, inner, lower lateral | 7/9 | 7.63 | 0.92 | 7.80 | 6.00 | 8.60 |
| li, inner, cervical | 7/7 | 6.81 | 0.95 | 6.70 | 5.30 | 8.10 |
| li, central, upper lateral | 7/13 | 10.93 | 0.60 | 11.00 | 10.00 | 11.80 |
| li, central, mid-lateral | 7/13 | 10.00 | 1.27 | 10.30 | 7.70 | 11.40 |
| li, central, lower lateral | 7/11 | 10.14 | 1.07 | 10.10 | 8.20 | 11.20 |
| li, central, cervical | 6/6 | 9.42 | 0.37 | 9.50 | 8.80 | 9.80 |
| li, outer, upper lateral | 7/11 | 12.67 | 0.96 | 12.80 | 11.00 | 13.70 |
| li, outer, mid-lateral | 7/12 | 12.51 | 1.36 | 12.10 | 10.50 | 14.60 |
| li, outer, lower lateral | 7/13 | 11.91 | 1.48 | 12.30 | 9.00 | 13.10 |
| li, outer, cervical | 7/10 | 11.07 | 1.63 | 11.00 | 8.40 | 13.10 |

TABLE S2

Significance of differences in enamel daily secretion rate (DSR) among different enamel areas (inner, central and outer third) of buccal and lingual crown regions of red fox M_1_. *P*-values < 0.05 are given in bold.

| Crown side | Crown region | Significance of differences among enamel areas (inner, central, outer third)^1^ | Pairwise comparisons^2^ | Nominal *p*-value/adjusted *p*-value^3^ |
| --- | --- | --- | --- | --- |
| buccal | upper lateral | ***p* = 0.002** | inner *vs* central | **0.028**/0.084 |
|  |  |  | inner *vs* outer | **0.028**/0.084 |
|  |  |  | central *vs* outer | **0.012/0.036** |
|  | midlateral | ***p* < 0.001** | inner *vs* central | **0.012/0.036** |
|  |  |  | inner *vs* outer | **0.012/0.036** |
|  |  |  | central *vs* outer | **0.012/0.036** |
|  | lower lateral | ***p* < 0.001** | inner *vs* central | **0.012/0.036** |
|  |  |  | inner *vs* outer | **0.012/0.036** |
|  |  |  | central *vs* outer | **0.012/0.036** |
|  | cervical | ***p* < 0.001** | inner *vs* central | **0.012/0.036** |
|  |  |  | inner *vs* outer | **0.012/0.036** |
|  |  |  | central *vs* outer | **0.012/0.036** |
| lingual | upper lateral | ***p* = 0.007** | inner *vs* central | **0.043**/0.129 |
|  |  |  | inner *vs* outer | **0.043**/0.129 |
|  |  |  | central *vs* outer | **0.018**/0.054 |
|  | midlateral | ***p* < 0.001** | inner *vs* central | **0.018**/0.054 |
|  |  |  | inner *vs* outer | **0.018**/0.054 |
|  |  |  | central *vs* outer | **0.018**/0.054 |
|  | lower lateral | ***p* < 0.001** | inner *vs* central | **0.018**/0.054 |
|  |  |  | inner *vs* outer | **0.018**/0.054 |
|  |  |  | central *vs* outer | **0.018**/0.054 |
|  | cervical | ***p* = 0.002** | inner *vs* central | **0.028**/0.084 |
|  |  |  | inner *vs* outer | **0.018**/0.054 |
|  |  |  | central *vs* outer | **0.028**/0.084 |

^1^Friedman ANOVA by ranks

^2^Wilcoxon matched pairs test

^3^Bonferroni adjusted *p*-value

TABLE S3

Linear enamel thickness (LET, µm) and ameloblast secretory lifespan (ASL, days) for different crown regions of red fox left M_1_, bu – buccal, li – lingual, *n* – number of teeth for which data were obtained.

| Variable/crown region | *n* | Mean | SD | Median | Minimum | Maximum |
| --- | --- | --- | --- | --- | --- | --- |
| LET, bu, upper lateral | 8 | 421.4 | 23.2 | 430.0 | 380.0 | 443.0 |
| LET, bu, mid-lateral | 8 | 441.1 | 11.3 | 438.5 | 430.0 | 467.0 |
| LET, bu, lower lateral | 8 | 425.5 | 26.0 | 427.5 | 380.0 | 454.0 |
| LET, bu, cervical | 8 | 353.9 | 31.0 | 360.5 | 311.0 | 399.0 |
| LET, li, upper lateral | 7 | 374.0 | 34.0 | 377.0 | 320.0 | 417.0 |
| LET, li, mid-lateral | 7 | 304.0 | 64.6 | 291.0 | 219.0 | 399.0 |
| LET, li, lower lateral | 7 | 247.7 | 46.8 | 245.0 | 177.0 | 323.0 |
| LET, li, cervical | 7 | 190.7 | 39.3 | 194.0 | 122.0 | 240.0 |
| ASL, bu, upper lateral | 8 | 34.6 | 2.6 | 33.0 | 32.0 | 38.0 |
| ASL, bu, mid-lateral | 8 | 35.8 | 2.0 | 36.0 | 33.0 | 39.0 |
| ASL, bu, lower lateral | 8 | 33.6 | 3.5 | 32.5 | 30.0 | 39.0 |
| ASL, bu, cervical | 8 | 29.4 | 3.4 | 29.0 | 25.0 | 36.0 |
| ASL, li, upper lateral | 6 | 34.5 | 3.2 | 35.5 | 29.0 | 37.0 |
| ASL, li, mid-lateral | 7 | 26.9 | 4.4 | 25.0 | 21.0 | 33.0 |
| ASL, li, lower lateral | 7 | 22.6 | 3.7 | 23.0 | 17.0 | 28.0 |
| ASL, li, cervical | 7 | 18.1 | 4.5 | 17.0 | 11.0 | 25.0 |

TABLE S4

Significance of differences in linear enamel thickness (LET) among the four different crown regions (upper lateral, mid-lateral, lower lateral, cervical) of red fox M_1_. *P*-values < 0.05 are given in bold.

| Crown side | Significance of differences among the 4 crown regions^1^ | Pairwise comparisons^2^ | Nominal *p*-value/adjusted *p*-value^3^ |
| --- | --- | --- | --- |
| buccal | ***p* < 0.001** | upper lateral *vs* mid-lateral | 0.059/0.354 |
|  |  | upper lateral *vs* lower lateral | 0.674/1 |
|  |  | upper lateral *vs* cervical | **0.012**/0.072 |
|  |  | mid-lateral *vs* lower lateral | 0.107/0.642 |
|  |  | mid-lateral *vs* cervical | **0.012**/0.072 |
|  |  | lower lateral *vs* cervical | **0.012**/0.072 |
| lingual | ***p* < 0.001** | upper lateral *vs* mid-lateral | **0.018**/0.108 |
|  |  | upper lateral *vs* lower lateral | **0.018**/0.108 |
|  |  | upper lateral *vs* cervical | **0.018**/0.108 |
|  |  | mid-lateral *vs* lower lateral | **0.018**/0.108 |
|  |  | mid-lateral *vs* cervical | **0.018**/0.108 |
|  |  | lower lateral *vs* cervical | **0.018**/0.108 |

^1^Friedman ANOVA by ranks

^2^Wilcoxon matched pairs test

^3^Bonferroni adjusted *p*-value

TABLE S5

Significance of differences in ameloblast secretory lifespan (ASL) among the four different crown regions (upper lateral, mid-lateral, lower lateral, cervical) of red fox M_1_. *P*-values < 0.05 are given in bold.

| Crown side | Significance of differences among the 4 crown regions^1^ | Pairwise comparisons^2^ | Nominal *p*-value/adjusted *p*-value^3^ |
| --- | --- | --- | --- |
| buccal | ***p* = 0.004** | upper lateral *vs* mid-lateral | **0.012**/0.072 |
|  |  | upper lateral *vs* lower lateral | **0.012**/0.072 |
|  |  | upper lateral *vs* cervical | **0.012**/0.072 |
|  |  | mid-lateral *vs* lower lateral | 0.076/0.456 |
|  |  | mid-lateral *vs* cervical | **0.018**/0.108 |
|  |  | lower lateral *vs* cervical | **0.012**/0.072 |
| lingual | ***p* = 0.001** | upper lateral *vs* mid-lateral | **0.028**/0.168 |
|  |  | upper lateral *vs* lower lateral | **0.028**/0.168 |
|  |  | upper lateral *vs* cervical | **0.028**/0.168 |
|  |  | mid-lateral *vs* lower lateral | 0.052/0.312 |
|  |  | mid-lateral *vs* cervical | **0.028**/0.168 |
|  |  | lower lateral *vs* cervical | **0.018**/0.108 |

^1^Friedman ANOVA by ranks

^2^Wilcoxon matched pairs test

^3^Bonferroni adjusted *p*-value

TABLE S6

Enamel extension rate (EER, µm/day) and enamel formation front angle (EFFa, degrees) in the buccal and lingual crown portions of red fox left M_1_, bu – buccal, li – lingual, *n* – number of teeth for which data were obtained.

| Crown region | *n* | Mean | SD | Median | Minimum | Maximum |
| --- | --- | --- | --- | --- | --- | --- |
| EER, bu, 1st (cuspal) third | 8 | 144.6 | 9.9 | 144.6 | 132.2 | 160.0 |
| EER, bu, 2nd (middle) third | 8 | 124.0 | 7.8 | 124.0 | 113.4 | 133.6 |
| EER, bu, 3rd (cervical) third | 8 | 85.0 | 9.1 | 85.8 | 70.0 | 100.0 |
| EER, li, 1st (cuspal) third | 7 | 170.3 | 18.7 | 158.8 | 150.3 | 195.3 |
| EER, li, 2nd (middle) third | 7 | 132.7 | 17.8 | 137.2 | 111.2 | 159.6 |
| EER, li, 3rd (cervical) third | 7 | 96.7 | 6.3 | 97.0 | 88.7 | 103.8 |
| EFFa, bu, upper | 8 | 4.06 | 0.28 | 4.02 | 3.61 | 4.51 |
| EFFa, bu, lower | 8 | 5.22 | 0.32 | 5.15 | 4.91 | 5.89 |
| EFFa, li, upper | 7 | 3.88 | 0.12 | 3.88 | 3.75 | 4.11 |
| EFFa, li, lower | 8 | 4.46 | 0.27 | 4.41 | 4.16 | 4.93 |
